# Critical Design Factors for Electrochemical Aptasensors Based on Target-Induced Conformational Changes: The Case of Small-Molecule Targets

**DOI:** 10.3390/bios12100816

**Published:** 2022-10-01

**Authors:** Andra Mihaela Onaş, Constanţa Dascălu, Matei D. Raicopol, Luisa Pilan

**Affiliations:** 1 Advanced Polymer Materials Group, University ‘Politehnica’ of Bucharest, 1-7 Gheorghe Polizu, District 1, 011061 Bucharest, Romania; 2 Faculty of Applied Sciences, University ‘Politehnica’ of Bucharest, 313 Splaiul Independenţei, District 6, 060042 Bucharest, Romania; 3 Faculty of Chemical Engineering and Biotechnologies, University ‘Politehnica’ of Bucharest, 1-7 Gheorghe Polizu, District 1, 011061 Bucharest, Romania

**Keywords:** nucleic acid aptamers, electrochemical aptasensor, small target molecules, conformational changes

## Abstract

Nucleic-acid aptamers consisting in single-stranded DNA oligonucleotides emerged as very promising biorecognition elements for electrochemical biosensors applied in various fields such as medicine, environmental, and food safety. Despite their outstanding features, such as high-binding affinity for a broad range of targets, high stability, low cost and ease of modification, numerous challenges had to be overcome from the aptamer selection process on the design of functioning biosensing devices. Moreover, in the case of small molecules such as metabolites, toxins, drugs, etc., obtaining efficient binding aptamer sequences proved a challenging task given their small molecular surface and limited interactions between their functional groups and aptamer sequences. Thus, establishing consistent evaluation standards for aptamer affinity is crucial for the success of these aptamers in biosensing applications. In this context, this article will give an overview on the thermodynamic and structural aspects of the aptamer-target interaction, its specificity and selectivity, and will also highlight the current methods employed for determining the aptamer-binding affinity and the structural characterization of the aptamer-target complex. The critical aspects regarding the generation of aptamer-modified electrodes suitable for electrochemical sensing, such as appropriate bioreceptor immobilization strategy and experimental conditions which facilitate a convenient anchoring and stability of the aptamer, are also discussed. The review also summarizes some effective small molecule aptasensing platforms from the recent literature.

## 1. Introduction

Over the last decades, biosensor technology has been proving its capability to complement or replace many of the complicated, relatively expensive, and time-consuming traditional analytical methods [1,2,3,4]. More recently, the use of nucleic acid aptamers as biorecognition elements has brought numerous advantages in comparison with the enzymes or antibodies commonly used in biosensors, such as smaller size, better stability, reversible binding ability, simple chemical modification, and a lower cost [4,5,6,7]. Moreover, unlike antibodies, aptamers can be obtained through chemical synthesis without batch-to-batch variation and free of microbial contamination [8,9,10]. The high specificity of aptamers for their molecular targets and their versatility for different types of analytical platforms has stimulated the development of a broad variety of biosensors based on aptamers (aptasensors) for domains such as medicine, environmental monitoring, or food safety [1,2,11]. In particular, the important progress observed in the electrochemical aptasensors’ development could be assigned to the simplicity and degree of control of the electrochemical transduction methods, the ease of aptamers’ integration in the electrochemical platforms, and the possibility to achieve ultrasensitive detection at a lower cost [11,12].

The aptamers with affinity for a desired target are selected in vitro from a random sequence library through an iterative method known as Systematic Evolution of Ligands by Exponential enrichment (SELEX) [13,14]. The method succeeded in isolating high-affinity and selective bioreceptors towards a large range of analytes, from ions, small molecules and macromolecules such as proteins, to complex targets such as bacteria. Particular emphasis has been placed on the isolation of aptamers that bind small molecules, considering that antibodies proved to be generally less effective for their detection [15]. However, the size of these molecules and the limited number of functional groups available for binding complicate the aptamer screening process [16,17]. The low affinity and specificity of many of the isolated aptamers for their small molecule targets induced weak recognition events in biosensing applications, and therefore a prior validation of the aptamer affinity through consistent evaluation standards is essential [4,5,18]. Alongside the aptamer selection step, the sensor design must also consider the proper incorporation of these molecules in the biorecognition layer [19]. The choice of an adequate immobilization strategy is important in respect with both the sensor performance, but also regarding basic requirements such as preserving the biological activity, stability, or the accessibility of the bioreceptor molecules [1,20]. The scope of this review was to provide a short overview on the challenges in selecting the aptamers with optimal binding properties for small molecules, and to offer guidance on choosing the appropriate solutions for designing electrochemical aptasensors for these types of targets. For the sake of brevity, our discussion is limited to the approaches that use the conformational changes of the aptamer to detect the binding events. An up-to-date overview of some relevant examples of electrochemical aptasensing platforms for small molecules, based on the originally-selected or engineered aptamers, classified according to the type of the detecting strategy, is also provided.

## 2. Aptamers as Tools for the Detection of Small Molecules 

Small molecules are low-molecular-weight organic compounds (<1000 Da) that include a variety of natural and synthetic compounds such as amino acids, sugars, fatty acids, phenolic compounds, alkaloids, nucleotides etc. [21]. The small-molecule sensors based on aptamers may serve as effective tools for their sensitive and accurate detection, providing important benefits in a broad range of domains from diagnostic and treatment monitoring to the detection of environmental and food contaminants [18,22].

Although the use of aptamers as biorecognition elements for developing sensors for small molecules was considered for the first time in the mid-1990s, the vast majority of works claiming the use of these detection platforms for small molecules detection have been developed after the 2000s [4]. So far, a significant number of aptamer sequences that bind to small molecules with high affinity and specificity have been described. However, for many relevant molecules no aptamers are yet available, or some of the isolated aptamers need to be further optimized as they do not have the required affinity or specificity for real-world applications [4,6,23]. The small size of the target and the lack of functional groups available for immobilization or for interaction with nucleic acids makes the selection process of small molecules’ aptamers very challenging [16,17]. Nevertheless, the current progress in SELEX technologies such as high-throughput sequencing (HTS) and post-SELEX optimization procedures has led to an improved screening of aptamers that are selective to small molecules [4,23,24].

Nucleic-acid aptamers have displayed a remarkably high degree of selectivity for their cognate targets, being able to recognize even minor structural differences. Although numerous examples of aptasensing strategies for small molecules detection have been reported, the large majority are proof-of-concept systems employing adenosine triphosphate (ATP) and cocaine-specific aptamers [25]. The RNA aptamer for theophylline represents another popular example, one of its interesting features being the very high binding affinity relative to caffeine (10,000-fold), although the latter differs from theophylline by a single methyl substituent on a nitrogen atom [26]. Also highly cited in the literature are the aptamers for citrulline and arginine [27], which show a low dissociation constant for their corresponding targets, but no detectable affinity for other amino acids [28]. Whereas the binding affinity is the primary parameter that defines the interaction between the aptamer and its target molecule, the structural features involved in the recognition event have a bigger impact for the design and the detection performance of the final sensor [29]. Ensuing the target recognition via different types of interactions (van der Waals interactions, hydrogen bonds, electrostatic attraction, etc.) [16], the aptamers can exhibit a large diversity of structural patterns, their folding properties being exploited in the design of sensors. Typical secondary structures that can be induced by binding with a target molecule are stem-loops (hairpins), three-way junctions, or G-quadruplexes [16,30,31,32]. The recognition event is further translated into an electrochemical signal by employing both label-free and labeled strategies. Label-free strategies rely on changes that occur in the electrochemical interface after the aptamer-target binding, that can be evaluated by EIS, or on the intrinsic redox signal of the bound-target molecule [33,34]. The label-based strategies involve either aptamers or complementary sequences tagged with redox-active moieties or soluble electroactive molecules that interact diffusively with the aptamers [34,35] The detection mechanisms for label-based strategies include target binding-induced conformational changes of aptamers [36,37], target binding-induced aptamer or complementary strand displacement [38], and the adoption of sandwich assays that include a second recognition element (aptamer or antibody) labeled with enzymes, metal nanoparticles, etc. [34,39]. Aptasensors based on conformational changes upon target analyte binding are considered one of the most simple and efficient devices, as they do not need multiple steps in the transduction event which facilitates the fabrication of reusable biosensors [35].

## 3. The Generation of Aptamers for Small Molecules

### 3.1. General Aspects of the SELEX Process

The development of aptamers and the associated SELEX technologies have gained enormous attention over the past 30 years. The SELEX process, first reported in 1990, is employed for screening the sequences that specifically bind a particular target or ligand from very large libraries of oligonucleotides (either single-stranded DNA or RNA) [40]. It consists of an iterative in vitro selection process, with the typical SELEX cycle including successive steps such as binding (target molecule is incubated with a random library), partitioning (the separation of the target-bound aptamers by physical or chemical methods), and amplification of the selected sequences [40,41,42]. The oligonucleotides selected and enriched during several SELEX cycles are further analyzed by cloning and sequencing individual clones. Ceasing the selection process at the right stage is another important element, as this can influence the expended costs and time; performing the selection beyond a critical point increases the probability of enriching nonspecific aptamers [41]. Therefore, multiple validation assays of the binding affinity are required to monitor the progress of the aptamer selection [41]. Over the past decades, improved variants of the originally developed SELEX techniques have been proposed, with the aim to either increase the efficacy of the process, to reduce the selection time, or to optimize aptamers in order to achieve new designs and beneficial features [43].

In this review we aim to bring clarity in respect to several critical aspects related to aptamer selection for small molecules when envisaging sensors’ application, so in the following we briefly provide an overview of the challenges and summarize the appropriateness of several SELEX methods for these targets. Selected biosensing applications presented in Section 7 of this review will confirm the feasibility of aptamers’ selection by these techniques.

### 3.2. Challenges and Limitations of the SELEX Process for Aptamers Binding Small Molecules

Although a multitude of aptamer-based small-molecule sensors have been designed until now, the challenges for sensitive and specific detection of these targets in real-world analytical contexts still exists [4,44]. These challenges emerge both from the difficulty associated with the selection process of small molecules’ aptamers, and from the complexity of developing sensing platforms with general applicability [44]. There are several factors contributing to the first shortcoming which are related to the size, the low number of functional groups, and the limited interactions occurring between the small-molecule targets and the aptamers [23]. The presence of certain functional groups on the target molecule that allow for coupling is necessary in the partitioning process, which involves immobilization on a solid matrix [18]. Moreover, even if the coupling is possible, some of the functional groups are used for the immobilization, further decreasing the possible interactions with the aptamer [23]. On the other side, the introduction of functional groups that facilitate immobilization alters the target structure, which can diminish the affinity considerably or lead to false-positive binding events [4,6,45]. The reason stems from the fact that the oligonucleotide library is now exposed to the functionalized target instead of the unmodified molecule, increasing the chance of isolating sequences that actually display affinity towards other parts of the system, such as the linker arms [18]. A definite prediction on the outcome of the aptamer selection process for a particular small-molecule target cannot be made, but the physicochemical properties of the target can provide an insight on the difficulty of isolating an aptamer with high affinity [4]. For instance, the separation of target-bound sequences from free aptamers, a key step in the SELEX process, is difficult to achieve via certain techniques such as capillary electrophoresis if molecules have a low molecular weight and lack charged functional groups [18]. Although the technique is very effective when used for proteins, the complex aptamer small-molecule target displays an insufficient change in electrophoretic mobility when compared with the unbound aptamer [4,46].

Improved versions of SELEX specifically tailored for the isolation of aptamers binding small molecules with high affinity have been developed in the past. A special variant known as Structure-Switching or Capture SELEX is widely used for the selection of DNA aptamers that bind to small molecules that cannot be immobilized on solid surfaces [42]. The method was first described in 2005 by Nutiu et al. [47] and then successfully applied in 2012 as Capture-SELEX by Stoltenburg et al. [48]. It consists in using a SELEX library that contains a docking sequence incorporated into a random region that enables hybridization with complementary (capture) oligonucleotides fixed on a solid matrix. The conformational change in the aptamer sequence upon binding the target in solution induces undocking, and thus the release of the oligonucleotides with affinity to the selected analyte [48,49]. Several reported shortcomings of the Capture-SELEX method are the exclusion of sequences that bind irreversibly to the support when target binding is not accompanied by suitable conformational changes or the isolation of false-positive sequences that are eluted because of their weak interaction with the capture oligonucleotides [43]. Also, the limitations related to the complexity of the equipment and the experimental conditions that need to be closely monitored during the screening process make this technique difficult to implement [42,43].

The SELEX methods involving the immobilization of either target or oligonucleotide library on different surfaces may limit the interaction between the two, so that SELEX methods based on freely interacting library and target in solution have been proposed [44]. In this regard, Park et al. employed the use of graphene oxide as a partitioning agent, as the interaction of this material with the freely ssDNA sequences is stronger than with the target-bound folded DNA [44,50].

Moreover, in order to increase the selectivity, the whole screening process can be combined with other approaches that eliminate oligonucleotide sequences that bind nonspecifically, i.e., to the immobilization matrix or structurally similar interfering compounds [4,6,51]. Early developed methods of this kind are negative SELEX, introduced by Ellington and Szostak in 1992 [14], and the counter-SELEX method introduced by Jenison et al. in 1994 [26]. For example, Polisky et al. were able to isolate the aptamer for theophylline with 10,000 times greater affinity relative to caffeine by applying a counter-SELEX procedure [26]. Similarly, the SELEX process can be designed to isolate aptamers with high cross-reactivity for a series of structurally similar target species [4]. Toggle-SELEX could be successfully applied for the selection of cross-reactive aptamers for protein and cell targets, but proved to be very challenging when dealing with small-molecule targets. A relevant example is the attempt of the Stockley group to isolate aptamers with a broader specificity for the entire class of aminoglycosides antibiotics [52]. More recently, an improved variant developed by Xiao’s group, known as “parallel-and-serial” SELEX, proved to be successful in isolating class-specific aptamers for small molecules with a familial molecular core structure [53]. An additional counter-SELEX procedure allowed the isolation of an aptamer that bound with nanomolar affinities to 12 synthetic cathinones, members of the same drug family, and showed no affinity to other 11 compounds displaying a similar structure [53]. Recently, the same group proposed an algorithm that included all these SELEX procedures (conventional, counter, and “parallel-and-serial”) to facilitate the selection of high-quality aptamers for small molecules [54]. Based on the proposed algorithm, the authors were able to isolate from natural DNA libraries aptamers with nanomolar affinity for several cannabinoids, widely recognized as challenging targets [54].

The integration of high-throughput sequencing (HTS) with SELEX represents a very attractive approach, allowing a better assessment of the selection efficiency between rounds and the identification of sequences that are rare but show enhanced target-binding properties, and which may simply be missed by the conventional sequencing methods [55,56]. HTS allows for quantitative determination of the individual sequences in the pool and also their enrichment throughout successive rounds, so it can reveal different problems in the aptamer isolation process and help to conduct the SELEX process more efficiently [49,57]. The main drawbacks of HTS technology are its relatively high cost and the need to analyze a huge amount of generated data [57]. However, the application of HTS together with high-performance bioinformatics computing make the identification of aptamer candidates from the massive amount of enriched oligo sequences more feasible [57,58].

Other challenges appear in the context of adapting the aptamers obtained in solution for grafting on the surface of an electrode signal transducer, and this requires the post-engineering of aptamers obtained through SELEX [59]. More aspects regarding this approach are discussed in a following chapter.

## 4. Aptamer-Target Interactions—Affinity, Thermodynamic Parameters, and Structural Aspects 

### 4.1. The Binding Affinity Concept in the Context of Small Molecules Detection

The binding affinity, a main concept used to characterize aptamer-target interactions, is defined as the strength of the interaction between two or more molecules that bind reversibly [60]. The binding and the recognition of a target molecule by the aptamers is regulated by their three-dimensional shape and different types of interactions such as hydrophobic, electrostatic, van der Waals, hydrogen bonds, base stacking, as well as shape complementarity between the two molecules [16,61,62]. The binding affinity relates with the strength of such interactions and ultimately defines an aptamer’s performance. The estimation of the binding affinity between aptamers and their cognate targets predicts their potential use as biorecognition elements in analytical applications [62]. Generally, the binding affinity is estimated by measuring the association constant that is inversely proportional to the dissociation constant (K*_D_*) [61]. Oligonucleotides that exhibit strong interactions with their target, and thus have high binding affinities, are characterized by a low K*_D_*. Generally, the reported K*_D_* values are in the picomolar to nanomolar range for proteins, and weaker-affinity interactions with K*_D_* in the nanomolar to micromolar range have been reported for small molecules [63,64]. Other quantitative metrics associated with the binding activity are selectivity and specificity [25]. According to Eaton et al., a selection conducted for a high-affinity binding determines automatically a highly specific binding [17]. 

**Scheme 1:**
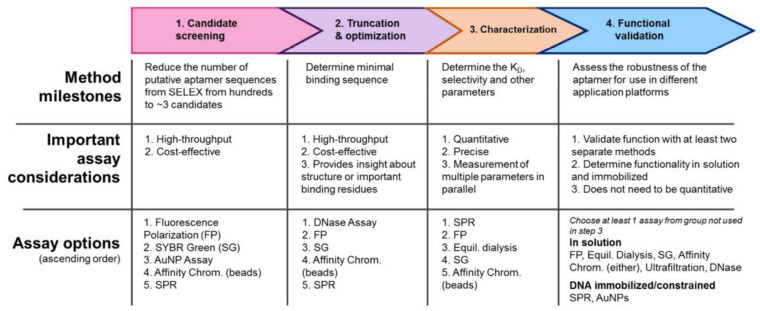
The workflow diagram proposed by McKeague et al. for the screening, truncation, characterization, and functional validation of a small molecule binding aptamer to ascertain an optimal integration into specific applications. Reprinted with permission from ref. [25]. Copyright 2015, American Chemical Society.

Although the prospects of generating a higher number of aptamer candidates for small molecules have improved with the latest advances in SELEX technologies, many sequences are poorly characterized and not always validated [65]. However, many research groups are emphasizing the requirement for adequate quality standards in order to certify the aptamers’ properties [56]. Choosing a series of aptamers for ochratoxin A (OTA), as a model of small-molecule target, McKeague et al. identified inconsistencies between the conventional assays of aptamer affinity, and illustrated the necessity for complementary characterization strategies that provide more quantitative metrics [25]. In this context, the group proposed an efficient and flexible methodology that facilitates a reliable screening, characterization and functional validation of small-molecule binding aptamers (Scheme 1) [25].

Beside affinity and selectivity, the determination of several other parameters such as buffer and ionic strength sensitivity should be performed when employing aptamers as bioreceptors, as it is known that experimental conditions such as buffer, ions, pH, temperature can influence the affinity of aptamers [64,66,67].

### 4.2. The Thermodynamics of the Aptamer-Target Interaction

The thermodynamic quantitative analysis of the aptamer-target complex formation under equilibrium conditions provides both the understanding of the interactions between the two entities, but also the base for improving these interactions [68]. From this perspective, the important parameters associated with the binding event are the change in Gibbs free energy (ΔG), the thermodynamic driving force of the aptamer-target complex formation, and the enthalpy, and entropy changes (ΔH, and ΔS respectively). The relationship between the three quantities for a process carried out at a temperature T is: ΔG = ΔH − T · ΔS. A greater negative ΔG value results in lower K*_D_* values and defines higher affinity aptamers and a more stable aptamer-target complex [69]. Generally, a negative value of ΔH, referred to as favorable ΔH, indicates an increased number of interactions between the aptamer and the target, whereas a positive value of ΔH is associated with a weaker interaction [62]. However, the enthalpy variation of in the aptamer-target binding process is not only related to the formation of noncovalent interactions between the two entities, but it is also influenced by the solvent, because it includes additional contributions arising from the disruption of hydrogen bonds and van der Waals interactions between the solvent and aptamer or target [16]. For example, according to Sakamoto et al., the aptamers with high affinity exhibit an enthalpy-driven binding [16]. The entropy change associated to the binding process comprises three terms that may introduce both favorable and unfavorable contributions to the total free energy of the process. The favorable contribution, meaning a large positive value of ΔS, results from an increase in the entropy of the surrounding water molecules during the recognition process. Nevertheless, a binding process induced by a large positive entropy change is usually associated with nonspecific interactions between aptamers and solvent molecules, which are characteristic for low-affinity aptamers [16,62]. The unfavorable contribution corresponds to entropic changes related to the decrease in the conformational entropy and the loss of the rotation and translation degrees of freedom of the aptamer-target complex in comparison with the free molecules [16,62]. Therefore, the intrinsic flexibility of an aptamer, which is very sensitive to its environment, causes an instability of its conformation, and thus a diminished affinity and selectivity for its specific target [8,64].

Another common effect, known as enthalpy-entropy compensation, appears when the aptamer-target binding is induced either by a favorable enthalpy change or a favorable entropy change, each combined with an entropy or an enthalpy drop, respectively. Understanding the thermodynamics of the binding process and identifying the actual binding mechanism are very important as, by carefully choosing experimental conditions or aptamer-structure modifications, one can optimize the enthalpic or entropic contribution to the total free energy [68]. Methods employed for measuring the binding affinity can be classified as direct methods, which do not require the physical separation of the aptamer-target complex from the free aptamers and target molecules, and separation-based methods [68]. No method is recognized as being universally applicable for small molecule aptamers, but several innovative approaches have been proposed in the last few years [18].

### 4.3. Methods for Determining the Binding Parameters of Aptamers and Challenges for Small-Molecule Targets

Methods for determining the dissociation constant are based on binding isotherm measurements. K*_D_* determination methods can either be immobilization-based (heterogenous), like surface plasmon resonance (SPR) or quartz crystal microbalance (QCM), or immobilization-free (homogenous), like isothermal titration calorimetry (ITC) or capillary electrophoresis (CE). When choosing the appropriate method for determining K*_D_*, one should consider the particularities of the methods and the target analyte. One of the major problems is the high variability of detected dissociation constants with different experimental methods [6,25]. Recent reports have emphasized the challenges for small-molecule targets [18,70], concluding that no determination method can be considered universally applicable in this case. For these reasons, the choice of the K*_D_* determination method should also consider the final application of the aptamer. For applications where the oligonucleotide is bound to a substrate, immobilization-based techniques are recommended, and for applications where the aptamer is used in a free environment, homogenous methods are more suitable.

**Figure 1:**
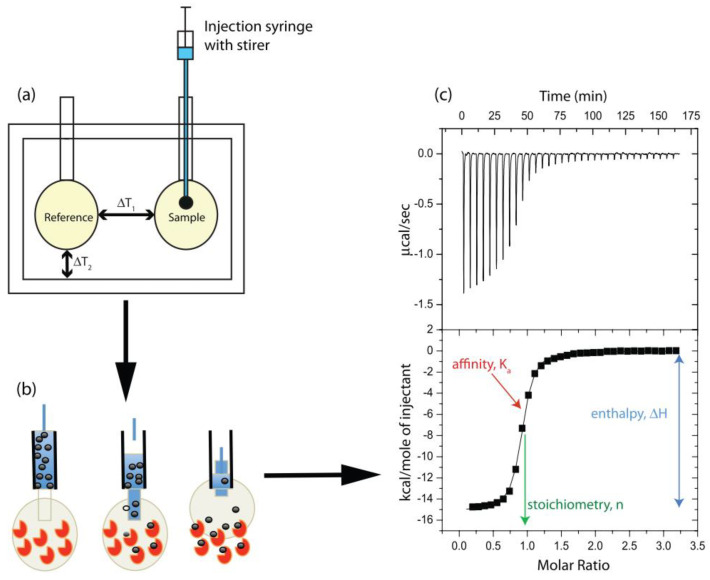
Schematic illustration of a typical ITC measurement (**a**) Scheme of a typical ITC instrument (∆T_1_, ∆T_2_—the temperature difference between the two cells, and the cell and adiabatic jacket, respectively, both are zero during an experiment); (**b**) Illustration of the binding process when target is injected into the cell containing aptamer. (**c**) Thermogram showing interaction of an aptamer with its target: (**top**) the raw titration data showing the heat resulting from each injection of the target into the aptamer solution; (**bottom**) the integrated heat (after subtraction of the heat of dilution) fitted to a 1:1 binding model (experimental data from [72], results: K*_D_* = 0.20 ± 0.05 µM, ∆H = −14 ± 1 kcal/mol, n = 0.98). Reproduced from [73] (open access).

Many studies report isothermal titration calorimetry (ITC) as the standard method for thermodynamic measurements. This technique provides most of the thermodynamic parameters characteristic for aptamer-target interactions: dissociation constant (K*_D_*), Gibbs free energy (ΔG), enthalpy change (ΔH), the entropy change (ΔS) and number of binding sites of the oligonucleotide (n) [71]. The method is based on measuring the temperature difference between a reference cell and a sample-containing cell, both inserted in an adiabatic jacket (Figure 1). 

ITC proved to be a powerful technique for the determination of optimal binding conditions. Neves et al. investigated the effect of pH and ionic strength upon the binding affinity of a cocaine aptamer, concluding that the optimum conditions are reached for pH 7.4 in the absence of sodium chloride [74]. Challier et. al. used ITC measurements for the determination of the binding affinity for an L-tyrosinamide specific aptamer, reporting a K*_D_* value of 4.9 μM [75]. This method has also been employed for the quantification of the quinine-binding affinity and thermodynamic parameters of a set of sequence variants for a cocaine-binding aptamer [31].

Microscale thermophoresis (MST) is based on the modification in solution diffusion rate of the bound aptamer-target complex when compared to the free aptamer [4]. Fluorescence must be exhibited either by the labeled aptamer or by the target in order to enable thermophoretic measurements. Baaske et al. were the first to use MST for ATP/AMP-binding aptamers as small-molecule specific oligonucleotides [76]. Using MST measurements, Rangel et. al. have reported K*_D_* values for ochratoxin A in the nM range, with the lowest value corresponding to a 31-nt aptamer (71 nM) [77].

Capillary electrophoresis (CE) is a separation-based method in which an electrical field is used for the separation of charged components by their molecular size [78]. For small molecule aptamers, this method is particularly challenging since the target-aptamer complex typically shows a small increase in size when compared to the free aptamer. Kinetic capillary electrophoresis has been recently proposed as a variant of CE in which there is no labeling requirement for the target analyte [79]. An automated version of the method, microchip CE, has also been employed for the rapid and low-cost determination of K*_D_* for aptamers that bind either small or large target analytes. Hu and Easley reported the use of an automated microchip CE protocol for an ATP-binding aptamer, obtaining a K*_D_* value of 71 μM at 30 °C [70].

The filter-binding assay is a homogenous method that requires prior radiolabeling of the aptamer for the determination of the binding ability [80]. For this protocol, a solution containing the labeled aptamer, target, and aptamer-target complex is filtered in order to remove the complex, and the radioactivity of the filtrate is measured. The dissociation-constant value is expressed as concentration of the unbound complex at half-maximum bound-fraction value [81]. Donaldson et al. proposed a variation of this method called Differential Radial Capillary Action of Ligand Assay (DRaCALA), which can be suitable for small molecule aptamers [82].

Fluorescence-based methods rely on fluorescence intensity or polarization changes upon target-binding. For fluorescence intensity (FI) assays, the interaction between the two components may either increase or quench the fluorescence intensity. Labeling of the small molecule may be necessary if the molecule does not have intrinsic fluorescence [18]. Fluorescence polarization (FP) enables thermodynamic measurements by monitoring changes in polarization state of the emitted light as a result of target capture by the aptamer [26]. Molecules that possess intrinsic fluorescence are more suitable for this method. For example, Okazawa et al. have reported affinity studies of aptamers towards porphyrins [83]. Rangel et al. have also exploited the intrinsic fluorescence of ochratoxin A and its analogues for FP measurements [77]. FP measurements were employed by several other groups for K*_D_* determination: McKeague et al. reported K*_D_* values in the nM range for two highly specific ochratoxin A aptamers [25], Challier et al. reported a 0.44 μM dissociation constant for a 23-mer L-tyrosinamide aptamer [75], and Kwon et al. obtained a K*_D_* of 181 nM for a Kanamycin B-specific RNA sequence. The latter group also reported the K*_D_* values for several aminoglycoside aptamers, all of which were determined using FA [84].

Surface plasmon resonance (SPR) represents a powerful technique used for the characterization of molecular interactions at interfaces [85]. It is a heterogenous, label-free technique which enables real-time measurements. The main binding parameters can be determined through SPR: affinity and dissociation constants, kinetic rate constants, analyte concentration, and even stoichiometry [71]. This method exploits the change in refractive index that accompanies the formation of an aptamer-target complex. Either the oligonucleotide or the analyte is bound to a substrate, in the former case the biggest limitation being the small variation of the refractive index, leading to a decreased sensitivity of the technique. In the latter case, the biggest limitation is the decrease in aptamer-binding capacity. Although this method is known for its applicability to proteins as target analytes [18], it has also been used successfully for aptamers that bind small molecules such as codeine [86] and L-tyrosinamide [85].

The quartz crystal microbalance with dissipation monitoring (QCM-D) is a surface-sensitive technique that relies on the resonance-frequency shift of piezoelectric crystals as a result of a change in mass. Surface-molecule interactions such as adsorption or binding events lead to mass changes and thus, changes in resonance frequencies. Energy dissipation shifts are correlated with molecular structural variations upon target-binding [85]. Because small-molecule targets have low molecular weight, QCM-D measurements may not be considered suitable for the determination of aptamer-binding parameters [49]. Despite this inconvenience, Challier et al. successfully employed this technique for determining the binding affinity of L-tyrosinamide-binding oligonucleotides, yielding a dissociation-constant value of 3 μM [75]. Similarly, Özalp obtained a K*_D_* value of 49 μM for an ATP-specific aptamer [87].

### 4.4. Target-Induced Conformational Changes in Aptamers: Secondary 3D Structures Exploited in Electrochemical Sensors

A specific and significant structural change of the aptamer in the presence of its target that can generate a measurable and reliable transduction signal is a prerequisite for the application of aptamers in small-molecule detection [29]. In this context, several structural analysis methods can provide valuable details regarding the specific recognition of the target molecule by aptamers. Generally speaking, the discrimination mechanism involves changes in the secondary structure of aptamer molecules in the presence of the target, leading to optimized intermolecular interactions, and thus high affinity and specificity for the analyte [88,89]. Therefore, understanding the biorecognition mechanism and identifying the binding domain are important for performing changes in the aptamer sequence that improve the performance of the prospective sensor. Such changes usually consist in truncation or addition of nucleotides [16,29].

**Figure 2:**
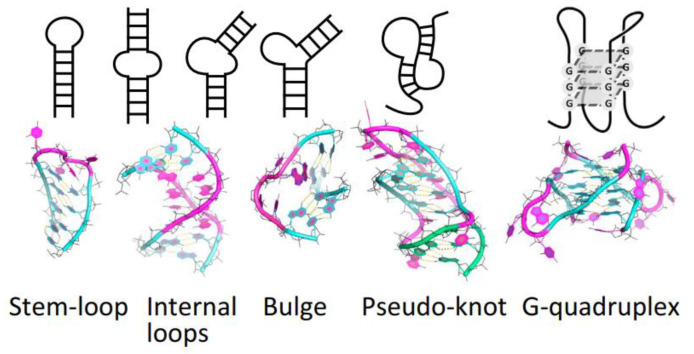
Secondary structural elements and motifs found in nucleic acids (above), and the representative three-dimensional structures for RNA from Protein Data Bank (aquamarine and green: nucleobases involved in base pairing or G-quadruplexes; magenta: other bases). Reproduced with permission from ref. [97]. Copyright 2022, Elsevier.

In the target-recognition process via various types of interactions, the aptamer molecules can adopt a large variety of structural patterns such as stem-loops, internal loops, bulges, pseudoknots, and G-quadruplexes (schematically depicted in Figure 2), which can be exploited for transduction [16,90,91,92,93,94,95]. These patterns arise frequently during in vitro selection of the aptamers, and are usually indicators of the potential binding sequences in the selected aptamers [96,97]. Most aptasensing platforms for small-molecule detection utilize structure-switching aptamers, which may adopt such a structural pattern or undergo other types of conformational change upon target-binding [98]. As many aptamers do not have such a built-in structure-switching functionality, many studies were devoted either to strategies for incorporating such a function into existing aptamers or to developing methods to obtain aptamers with intrinsic structure-switching functionality [44,99]. Many reports demonstrated that the thermodynamics of the switching process can influence sensing characteristics such as dynamic range and detection limit [100]. 

We will next discuss the stem-loop and G-quadruplex structural motifs, together with the typical features of the two classical examples of aptamers for small molecules: the aptamers for adenosine triphosphate (ATP) and cocaine.

#### 4.4.1. Stem-Loops

The first electrochemical biosensor based on binding-induced folding of a nucleic acid was developed in 2003 and consisted in a stable stem-loop DNA structure having one end covalently modified with a redox-reporting moiety, whereas the opposite end was modified with a long-chain alkane thiol, forming a self-assembled monolayer onto a gold substrate [101,102]. This biosensor was employed as a DNA hybridization sensor and functioned by inducing the unfolding of the stem-loop DNA probe in the presence of the complementary sequence that removed the redox group from the proximity of the electrode and suppressed the electrochemical signal. This approach was further generalized for various aptasensing platforms, and the employed strategies were divided in “signal-off”, when the interaction of a redox labeled aptamer with its target led to an increase in the distance between redox label and electrode, and the opposite case, the “signal-on” strategy, when the distance between redox label and electrode decreased upon target binding. Given the analogy between these sensors and the formerly developed fluorescent molecular beacons [103], they are commonly known as molecular aptamer beacons [104]. Therefore, the sensing mechanism of molecular aptamer beacons involved the denaturation of the stem structure, also called target-induced structure switching [104,105].

Different research works demonstrated that the binding affinity of previously identified stem-loop aptamers could be optimized by changing the stem-loop size, length or composition, and by performing mutations to better match the required analyte concentration range [106,107,108]. The SELEX protocol applied for a particular target molecule led to aptamers with a low K*_D_* value, usually suitable in a narrow concentration range when applied in sensing [108]. In this regard, Porchetta et al. established two general approaches for controlling the switching thermodynamics of the cocaine aptamer, in order to fine-tune its binding affinity and dynamic range over three orders of magnitude [106]. One of their strategies consisted in performing mutations at the distal site of the parent aptamer, leading to variants with lower switching-equilibrium constant and weaker overall affinity. The other one was based on designing oligonucleotides sequences pairing various regions of the aptamer, capable of stabilizing its nonbinding conformation and thus reducing the binding affinity [106]. Although the mutational approach can potentially lead to aptamers having different specificities from the original sequence, resulting in sensors with modified selectivity, in the latter approach the aptamer binding site remains unchanged, und so does the aptamer’s specificity [106]. Later, Armstrong and Strouse devised a method to modify the binding affinity of a previously isolated aptamer by incorporating its sequence into a stem-loop structure [108]. This strategy allowed them to tune the K*_D_* of the ATP aptamer previously isolated by Huizenga and Szostak [109] over several orders of magnitude. The advantage of inserting the sequence in a stem-loop structure over performing changes in the aptamer sequence is evident, as even single-site modifications in nucleic acids can randomly interfere in the secondary structure formation [108].

#### 4.4.2. Guanine-Quadruplex (G4)

A wide variety of aptamers that contain guanine-rich sequences are able to fold into secondary structures known as G-quadruplexes (G4) upon binding their target [95]. G4s are thermodynamically stable secondary structures of the guanine-rich nucleic acids. It was demonstrated that many G4s are more stable than double-stranded DNA, whereas their unfolding kinetics are much slower than that of DNA hairpin structures [110,111]. Although when initially discovered they were considered a structural curiosity, later studies have shown that G4 structures can be found in many important regions of the genome, such as the telomeric regions of chromosomes, and that they are involved in important biological processes such as the regulation of gene expression [92,111,112,113].

**Figure 3:**
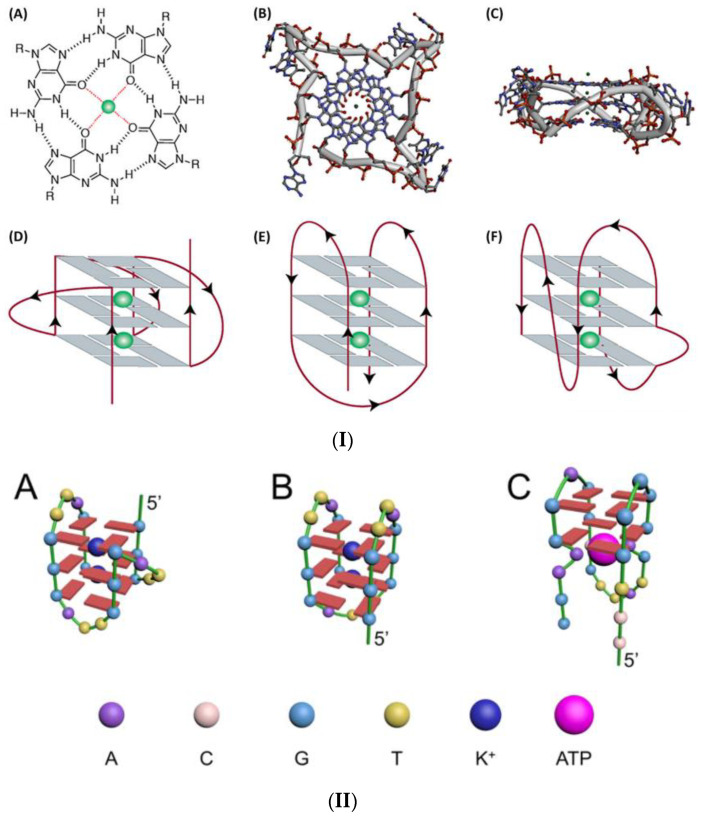
(**I**). G4 structure: (**A**) G-quartet with the central cation (green) coordinated to oxygen atoms; (**B**,**C**) crystal structure of human telomeric G4s (Protein Data Bank: 1KF1)—top and side view, respectively; (**D**–**F**) schematic illustration of G4s based on the strand orientation: (**D**) parallel, (**E**) antiparallel, and (**F**) hybrid with a bulge. Reprinted from [112] (open access); (**II**). (**A**) K^+^ aptamer: hybrid conformation, (**B**) K^+^ aptamer: anti-parallel conformation, and (**C**) ATP aptamer: anti-parallel conformation. Reprinted with permission from ref. [115]. Copyright 2021, American Chemical Society.

The structure of G4s consists of G-quartets in which guanine bases associated via Hoogsteen hydrogen bonds are stacked in a planar distribution, stabilized by the presence of centrally located monovalent cations (K^+^ and Na^+^) coordinated to oxygen atoms from the guanine carbonyl groups [111,112]. This arrangement explains why the formation of G4s is favored by the physiological buffer conditions [111]. Two or more G-quartets can associate through π–π-stacking interactions, and form higher-order G4 structures [114]. The G4 structures are polymorphic and, depending on the number of strands, can be classified as monomeric, dimeric, or tetrameric, when considering the adjacent strands’ orientation as parallel or antiparallel (Figure 3) [92].

G4-based aptasensors allowed the detection of a large variety of analytes, ranging from proteins and even cells to small molecules such as antibiotics, toxins, and metal ions [95]. A recent review summarizes several G-quadruplex-based assays for small molecules’ detection [114]. An illustrative example is the electronic nanoswitch developed by Wu et al., able to change reversibly its conformation between a rigid G4 structure and a flexible linear structure in the presence of K^+^ cations [116]. The approach employed a 41-nt DNA sequence that contained a single three-guanine region close to the 3′-end, a ferrocene group as the signaling tag at the 3′-end, and a thiol moiety at the 5′-end for immobilization at a substrate gold electrode. The structural transition between the flexible single-stranded structure and G4 structure produced variations in the distance between the redox tag and the electrode surface, that eventually determined a reversible change in the current intensity. In other words, a mechanical motion of the ssDNA molecule relative to the substrate could be transduced into an easily detectable electrochemical signal. Moreover, as K^+^ binds specifically to G4s, the platform could also serve as a reusable biosensor for the selective detection of potassium [116].

#### 4.4.3. ATP-Binding Aptamer

**Scheme 2:**
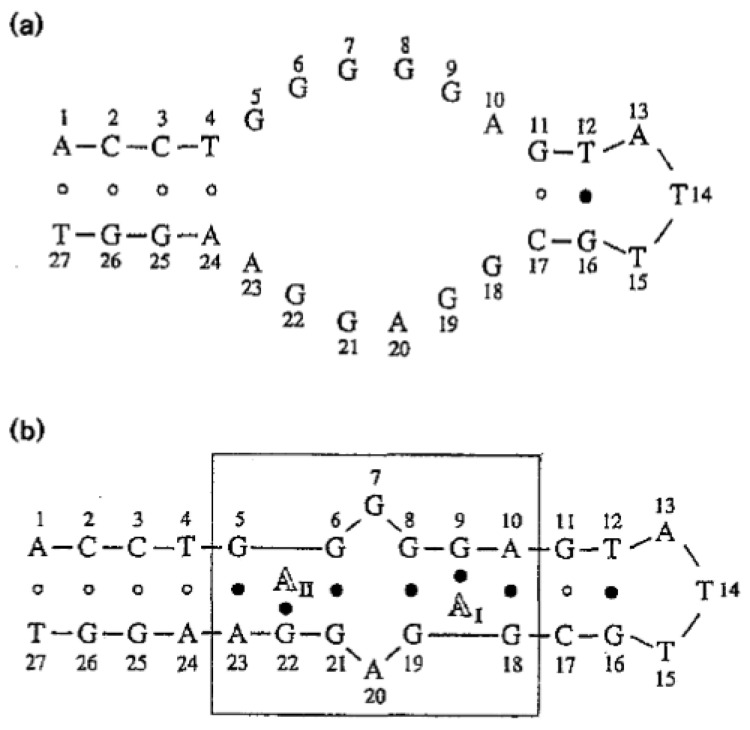
(**a**) The sequence and Watson-Crick pairings for the original 27-mer ATP-binding DNA aptamer selected by Huizenga and Szostak [109] (**b**) The secondary structures determined by Lin and Patel for the original ATP-binding aptamer. The two binding sites are designated A_I_ and A_II_. Reprinted with permission from ref. [119]. Copyright 2022, Elsevier.

As one of the most investigated small-target aptamers, the DNA aptamer for adenosine triphosphate (ATP) became widely used as a model for establishing new aptasensing strategies [6,117]. It was first selected by Huizenga and Szostak in 1995 [109], and then, independently, in the same year by Nutiu and Li [47]. The ATP-binding DNA aptamer is a structure-switching aptamer with the particularity of binding two target molecules via a positive cooperative binding mechanism [117,118]. The aptamer shows similar affinity, in the micromolar range, for other adenosine derivatives such as adenosine monophosphate (AMP), cyclic adenosine monophosphate (cAMP) or adenosine triphosphate (ATP), but it does not bind to other nucleosides [15]. Lin and Patel elucidated the structure of the ATP aptamer bound to two molecules of AMP using NMR spectroscopy (Scheme 2) [119]. Early structural investigations revealed that the unfolded ATP aptamer has a disordered region, which becomes ordered upon ligand binding [117,119]. Subsequent experimental and computational studies confirmed that this aptamer presents a random conformation at low ionic strength, but in the presence of its target or under high-ionic-strength conditions it switches to a rigid double-loop hairpin structure [120]. By employing NMR, ITC and thermal stability studies, Slavkovic et al. showed that the cooperative binding of the two target molecules is a result of a population-shift mechanism. Specifically, the binding event of the first target molecule stabilizes the poorly ordered structure of the free aptamer, and alters the binding sites and the region between them, inducing in this way a higher affinity-binding of the second molecule [117].

By employing the DNA aptamer for ATP, Zhang et al. studied the correlation between the number of binding sites and sensitivity, using a rational sequence design consisting in removing each binding site individually. The ITC results showed that single-pocket aptamers have a comparable binding affinity and specificity. Moreover, because the new single-site aptamer is non-cooperative, a sensor fabricated with this bioreceptor has a better sensitivity at lower concentrations of analyte [15].

Several electrochemical sensors based on this aptamer tried to exploit the G4 structure as a reporter system [115,121,122,123,124]. For example, Yu et al. developed an electrochemical platform based on porous anodic alumina (PAA) nanochannels for the quantitative detection of K^+^ ions and ATP molecules with good selectivity and reproducibility [115]. These authors immobilized the aptamers for both K^+^ and ATP (that contain G4 motifs) inside the silanized nanochannels of a PAA membrane. The presence of the target analytes induced the folding of both aptamers in the nanochannels into G4 structures, increasing the steric hindrance, as evidenced by a diminished anodic current of a redox indicator (hydroquinone solution) flowing through the channels.

#### 4.4.4. Cocaine-Quinine Competitive-Binding Aptamer

**Scheme 3:**
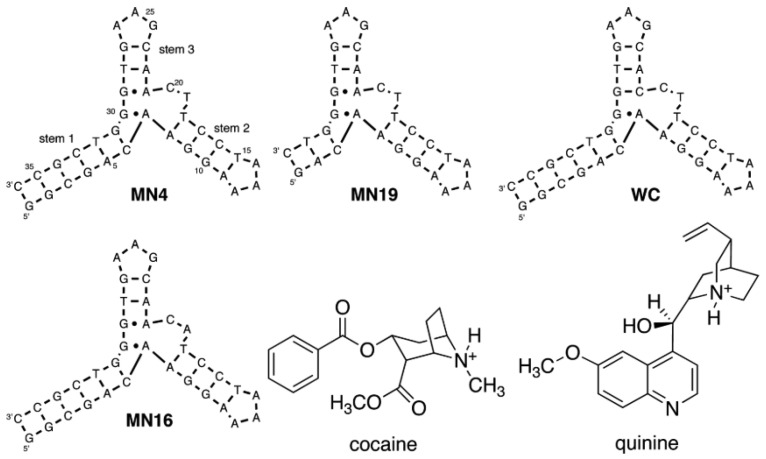
Sequences of several tested aptamers for cocaine and quinine targets. MN4 is an aptamer sequence that proved to bind cocaine a little tighter than the originally reported aptamer. MN4 is folded in the free state that does not change its secondary structure when binding cocaine or quinine. The other depicted sequences contain different modifications from MN4: three base pairs removed from stem 1 (MN19); A21 replaced with C at the three-way junction to restore the Watson−Crick pair (WC), and a weak-binding variant with T19 replaced by A (MN16). (dashes: Watson−Crick base pairs; dots: non-Watson−Crick base pairs). Reprinted with permission from ref. [31]. Copyright 2013, American Chemical Society.

Cocaine served as a representative target for testing new aptamer-based sensors due to the need for reliable and fast detection techniques in forensic analysis [125,126,127]. Also, numerous studies employing the cocaine aptamer exploited the structure-switching binding mechanism or target-induced folding that occurs upon target binding [72,128]. Different aptasensing strategies for the electrochemical detection of this small molecule were developed using the initial DNA aptamer developed by Stojanovic et al. (known as MNS-4.1 aptamer) [128] or one of its later variants [31,72,129]. The MNS-4.1 aptamer was reported as being highly selective for cocaine, and to have low affinity for the common cocaine metabolites. Its structure consisted in three stems built around a three-way junction, and it was presumed that one or more of these stems were unfolded in their free form, although all three are folded in the target’s presence [129]. Several variants of this aptamer have been investigated, some of which are presented in Scheme 3. Numerous studies on different variants of the original cocaine-binding aptamer performed by Johnson’s group led to several conclusions regarding structure-affinity relationships [31,72,74,129]. These authors showed that the stem length has an influence on target binding-shortening the stems decreases the binding affinity, and increasing the stem length leads to aptamers with increased affinity [74]. The same group revealed another interesting characteristic of this aptamer: it has an almost 30-fold stronger affinity for quinine than cocaine [31,72]. It was also shown that, although both quinine and cocaine bind at the same site on the aptamer, the existence of the bicyclic aromatic ring in quinine may favor stacking interactions [31,72]. For all targets that bind to this aptamer, the association is sustained by a negative enthalpy (ΔH = −11…−23.5 kcal mol^−1^), beside an unfavorable binding entropy compensation effect (−TΔS = 0.7…14 kcal mol^−1^) [72]. 

Subsequent affinity studies performed by Sachan et al. on the initial cocaine aptamer demonstrated that MNS-4.1 also binds some of the cocaine metabolites and aminoglycosides, with similar or higher affinities [130]. Interestingly, circular dichroism and fluorescence measurements did not show any evidence of large structural rearrangement of the cocaine aptamer upon target binding, and it was concluded that the aptamer specificity must be the consequence of its interaction with all faces of the cocaine molecule [130].

### 4.5. Methods for Determining the Structure of Aptamer-Small-Molecule Target Complexes

A necessary prerequisite for understanding aptamer binding mechanisms is obtaining adequate structural information regarding the aptamer, as well as the conformational changes that occur as a result of target binding [131]. Several characterization methods are commonly employed for this purpose, including circular dichroism, nuclear magnetic resonance, or X-ray crystallography. In many cases, more reliable results are obtained when experimental methods are employed in conjunction with molecular modeling.

Circular dichroism (CD) is a spectroscopic technique based on the differential absorption of left and right circularly polarized light by an optically active sample. CD is extremely valuable in biomolecule conformation studies so it can be used to investigate the interactions of aptamers with small-molecule targets. Oligonucleotide chains have a UV absorption region between 200 and 320 nm [4] and structure determinations are usually made by recording a CD spectrum of a sample and comparing it to a spectral library of biomolecules with known structures. Kypr et al. reported the use of CD in structural studies of DNA motifs, transitions, and denaturation processes [132]. Its use in target-biomolecule interaction has also been demonstrated by Ranjbar and Gill [133], and Rowe et al. [134] successfully used CD to investigate the structural modification of a RNA aptamer upon its interaction with tobramycin. Challenges in CD measurements include possible UV absorption of the target molecule, limited resolution, environmental factors (temperature, pH, ionic strength). 

Nuclear magnetic resonance (NMR) is perhaps the most useful structure determination method for aptamers, and two-dimensional NMR coupled with molecular dynamics simulations can provide information on atomic distances and bond angles, generating a three-dimensional model of the aptamer sequence [4]. Because NMR has a low sensitivity, rather high quantities of high-purity oligonucleotides are required, so short oligonucleotide sequences are preferred for this method to make it cost-efficient. 

For example, Neves at al. successfully used NMR to elucidate the structure of the cocaine-binding aptamer [129], and investigated the aptamer folding upon interaction with its target by examining the chemical shifts differences that appear after binding. A similar structural change was demonstrated by Slavkovic et al. for an ATP-specific DNA sequence [117].

X-ray crystallography uses monocrystalline samples for the determination of diffraction patterns, providing information about the spatial arrangement of atoms [4]. Compared to NMR, this technique provides higher resolution, but its major limitation is that measurements require single-crystal samples, which can be difficult to prepare. Moreover, the crystal structures of aptamers and their target complexes can be very different from their structures in solution. On the other hand, there is no limitation regarding the length of oligonucleotides. For these reasons, X-ray crystallography is considered more suitable for aptamers that bind bigger targets, such as proteins [135].

## 5. Aspects Related to Post-SELEX Optimization of the Aptamers

The post-SELEX optimization of a certain aptamer aims to improve one or more of its properties such as affinity, nuclease resistance, or thermal stability [136]. Usually, the optimization process consists of truncation, if the initial sequence includes unnecessary nucleotides that have no role in the interaction with the target molecule. The inconvenience is that the post-SELEX modification may disrupt the folded spatial structure of the aptamers and thus alter their binding properties and specificity. Therefore, identifying the sequence region critical for target binding is an important prerequisite for obtaining effective aptamers [137].

### 5.1. Challenges Related to Truncation of Aptamer Sequences

The elimination of unnecessary nucleotides by truncation usually has the role of reducing the production cost of the aptamers or to select minimal binding patterns for facilitating biosensor design [96,138]. Although minimizing the size of an aptamer can bring advantages, any modifications that alter the aptamer-target interactions may reduce the affinity, the specificity, and the stability of the bioreceptor [24]. Therefore, the truncation procedure needs to include the prediction of the secondary structure by computational tools, and the quantitative characterization and functional validation of the resulting new sequence [24,25,96].

It is assumed in general that the primer-binding regions flanking the random section of oligonucleotides library, involved in the amplification steps in conventional SELEX, and then part of the selected sequence, may cause non-specific binding or interfere with target-aptamer interactions [61,139]. Numerous studies demonstrated that these flanking sequences are difficult to remove post-SELEX, due to adjacent motifs or general destabilization of the aptamer structure [61]. For example, it has recently been demonstrated that a truncated aptamer for chloramphenicol does not actually bind to its target, although it has been employed in numerous other studies since its initial report in the literature [138]. The originally selected aptamer contained 80 nucleotides and was predicted to fold in a structure with two hairpins joined by a long loop [140]. The truncated sequence of only 40 nucleotides was obtained by the simple deletion of the 20-nucleotide constant primer binding sites from both the 3′ and 5′ ends, without further investigating its binding properties [141]. Tao et al. compared the predicted secondary structures of the initial and truncated sequences and showed that the truncation fully disrupted the hairpins, leading to a completely different secondary structure, with minimal probability to preserve the binding mechanism of the original aptamer. Moreover, the ITC, fluorescence spectroscopy, and AuNP-based label-free assays could not validate the binding of chloramphenicol to the truncated aptamer [138]. A similar debate was opened by Zara et al. [3], who investigated several previously selected organophosphorous-pesticide aptamers that did not recognize the specific analytes, even if micromolar dissociation constants have been reported in the referenced papers [142]. All these results demonstrate that it is very important to validate the binding properties of newly selected aptamers. Furthermore, for small-molecule aptamers the need for complementary characterization techniques involving homogeneous methods, to avoid surface effects, is very important [3]. This viewpoint has also been emphasized in a previous section that included the guidelines proposed by McKeague et al. to ensure a robust screening and functional validation of newly selected or truncated aptamers [25].

### 5.2. Post-SELEX Engineering Processes which Increase the Affinity of Aptamers

Many aptamer-based assays for real world applications require a fine tuning of the sensitivity, which can be accomplished through an affinity enhancement or attenuation via post-SELEX sequence engineering [44]. It was demonstrated that the SELEX technology may sometimes exclude high-quality aptamer candidates and cannot always deliver aptamers with optimal affinity and specificity for the intended application [143]. Although post-engineering modifications can also envisage the enhancement of other aptamers’ properties such as nuclease resistance, or thermal stability [136], in this section we will discuss only the strategies related to affinity tuning.

In Section 4.4.1. we presented several ways to modulate the binding affinity of the cocaine and ATP aptamers, which successfully led to an extension of the aptasensor dynamic range [106,108]. The employed strategies consisted in altering the aptamer thermodynamics by mutation approaches [106], or by the controlled incorporation of the aptamer in stem-loop architectures [108].

Other works reported truncation approaches that improved the binding affinity of aptamers, especially by deleting the fixed primer binding sequences, which presumably were not involved in binding [43]. Several papers reported a similar or even higher binding affinity and specificity when compared with the original selected aptamer [41]. For example, Kwon et al. performed the truncation of the originally developed 76nt DNA aptamers for oxytetracycline (OTC) [144] to an 8nt DNA aptamer having a high affinity and selectivity for the target [145]. The truncated sequence, that conserved only the original binding site and 6 additional specific bases, presented a higher binding affinity for OTC (K*_D_* = 1.067 nM) than the parent aptamer (K*_D_* = 9–121 nM). Moreover, the truncated aptamer demonstrated 500-fold higher sensitivity for tetracyclines detection, and a LOD of 0.1 nM [145].

The aptamers obtained through SELEX are sometimes fully folded in their free state, so that the generation of a conformational change upon target binding is not possible [72]. The introduction of a structure-switching functionality through truncation is complex and requires trials at different regions of the full-length aptamer [4,69]. In order to optimize a structure-switching aptamer for cocaine, Johnson’s group examined 24 truncated or mutated sequences of the original aptamer. By ITC and NMR analysis they succeeded to identify the target-binding domain and the sections critical for the secondary structure [74].

Computational or molecular docking is a simulation strategy that proved to be very efficient for the screening of a large set of targets and assessing the structures of aptamer-target complexes [69]. It can be applied between a small molecule that is flexible and a rigid macromolecule to predict the efficiency of the truncation procedure [69].

Xiao’s group proposed a general methodology to be applied for introducing structure-switching functionalities into the aptamers for small molecules, after discovering that by binding the target to its aptamer the nuclease digestion is inhibited a few nucleotides prior to the target-binding domain. This nuclease-directed truncation employs the exonuclease III (Exo III) enzyme, which completely degrades aptamers in their target’s absence [98]. As proof of concept, the researchers performed the digestion of the fully folded cocaine and ATP aptamers (having a three-way junction, and a stem-loop structure, respectively) using Exo III. The digestion product exhibited structure-switching functionality, and also preserved its target-binding affinity, making it suitable for sensing applications [98]. The same group has recently reviewed a large variety of approaches to develop aptamer-based sensors by employing fully folded aptamers [4].

Biniuri et al. proposed another interesting scenario to improve the binding features of the aptamers for their targets, that consisted in the covalent tethering of the redox-active methylene blue (MB) molecules to the aptamer chain [120]. The redox-active groups had the role of switching the binding properties of the aptamers for its target (ATP molecule) through oxidation and reduction steps. In this way the aptamers containing the MB-oxidized form showed high affinity toward ATP, although the aptamers tethering the reduced MB had no affinity for the target. The computational simulations showed that these flexible binding features of the aptamer were promoted by the intercalation of oxidized MB in the aptamer structure and the cooperative role of the H-bond stabilization [120].

## 6. Challenges in Generating Aptamer-Modified Electrodes Suitable for Sensing

### 6.1. The Selection of the Aptamer Immobilization Strategy

It should be emphasized that the most important aspect to be considered for the immobilization of an aptamer at the electrode surface is to preserve its molecular recognition ability [146]. Upon immobilization, the aptamers become prone to conformational changes under the influence of various factors such as the charge of the electrode and the environmental conditions (temperature, pH, ionic strength, etc.) [68]. For example, it was established that the conformation of aptamers unfolded in their free state is strongly influenced by the electrode surface charge, although aptamers with a folded conformation in the free state have weaker electrostatic interactions with the charged electrodes [146]. Consequently, the immobilization step can have a profound influence on the aptamer-target binding events which involve conformational changes. 

The selection of the immobilization strategy must consider the type of aptamer, the electrode material, the analyte’s properties, and also the operating conditions of the final sensor [68]. This step in the sensor design may influence the binding affinity, the stability, and the accessibility of surface-confined aptamers, the non-specific adsorption, and finally the overall performance of the final sensor [1,68]. Among the different methods applied for aptamers’ immobilization at the electrode surface, the covalent binding is the most common. However, other strategies such as physical adsorption, entrapment in a polymeric matrix, cross-linking, and noncovalent coupling through electrostatic or affinity interactions are utilized [20]. Depending on the chosen method, the aptamer molecules can be conveniently functionalized by amino, azido, thiol or biotin groups which can be incorporated at the 3′ and/or 5′ ends during the selection process, to facilitate immobilization onto different substrates [1]. The inclusion of spacer groups adjacent to the attachment point, such as alkyl chains, polyethylene glycol, hexamethyldiamine or oligo-thymidine sequences, proved to improve the analyte binding [1,19,68].

Gold and carbon-based electrodes are usually selected as substrates for developing electrochemical aptasensors for small molecules, and the immobilization method must take into account the properties of both the electrode material and the aptamer. The well-known benefits of gold as substrate for immobilization are its insignificant adsorption properties and ease of modification with compact and highly ordered chemisorbed self-assembled monolayers (SAMs), although the carbon-based materials are preferred due to their low cost [146]. Nevertheless, the ideal substrate electrode material should not only allow an efficient immobilization of the bioreceptor oligonucleotides on its surface, but also favor a sensitive detection of the recognition event by ensuring a strong interaction between the aptamer and the target, and a negligible nonspecific adsorption [68].

The surface immobilization of aptamers can be performed through electrostatic interactions at activated surfaces. This procedure is very simple; it does not require cross-linking reagents or chemical modification of the bioreceptor molecules, but the developed sensors often lack stability [20].

The technique of self-assembling molecular monolayers onto metal surfaces is extremely versatile due to its simplicity of preparation procedure and the large variety of adsorbent-adsorbate combinations [147]. It is a widely employed method in the construction of electrochemical aptasensors, in which thiol-modified ssDNA receptor molecules are attached onto gold surfaces via S-Au bonds. In many cases, the aptamers are immobilized together with diluent molecules such as mercaptohexanol [146,148], forming mixed monolayers. This approach allows the fine-tuning of an optimal receptor density, and the reduction of steric interactions that could hinder target binding [149]. Beside the ability to attach multiple aptamers or functional layers (mixed SAMs), the SAM surfaces can be easily integrated with techniques such as SPR and QCM [68].

Covalent surface grafting provides a robust and stable immobilization, without biomolecule leakage, but usually involves tedious protocols [20]. In this approach the electrode surface is first derivatized with reactive moieties that can form covalent bonds with bioreceptor molecules. In the electrochemical biosensors’ context, the electrochemically assisted covalent modification of the electrode surface is usually selected due to the possibility to easily control the surface reaction, and thus improve the overall reproducibility of the sensor fabrication process [1]. For example, the electrochemical reduction of aryl diazonium salts became a well-established tool for biosensor development [7,150,151,152,153,154]. Despite several challenges such as controlling the surface composition when preparing mixed layers, diazonium electrografting is simple, versatile, and, unlike the self-assembly method, involves short preparation times and long-term stability [155]. At the same time, aryldiazonium grafting can be performed on many types of electrode materials, from carbon-based electrodes (glassy carbon, screen-printed carbon electrodes, carbon nanotubes, graphene, diamond etc.) to silicon, various metals and indium tin oxide surfaces [151,156,157,158,159]. The concurrent coupling of different aptamer bioreceptors for multiple analytes’ detection by employing the electrochemical grafting method has also been reported [160]. Moreover, it allowed the simultaneous grafting of functional groups with an antifouling role, in order to diminish nonspecific adsorption of interfering species and thus increase the selectivity of the sensing assays [1,161,162,163]. The applicability of this approach for different electrochemical aptasensors developed for monitoring food contaminants has been recently reviewed by our group [1]. Most commonly the electrochemical reduction of aryldiazonium salts is applied for grafting substrate electrode with carboxyphenyl moieties that can bind amino-modified aptamer molecules through EDC (1-Ethyl-3-(3-dimethylami nopropyl)-carbodiimide) coupling reactions [7,153,164]. Likewise, grafting of phenyl layers containing alkynyl substituents allows the coupling of azide-terminated aptamers through Cu(I) catalyzed “click” chemistry [165]. Other covalent attachment procedures involve the use of functionally modified surfaces, where the most encountered functionalities are amine, carboxylic acid and hydroxyl. For instance, amine-modified substrates allow several aptamer immobilization reactions that include modification with glutaraldehyde, carbonyldiimidazole, succinimidyl 4-(N-maleimidomethyl)cyclohexane-1-carboxylate or symmetrical diisothiocyanates used as bifunctional linkers [166]. Nevertheless, the use of any covalent attachment protocol should facilitate the sensor fabrication process and ensure reproducibility when delivering a high immobilization yield.

### 6.2. The Influence of the Aptamer Surface Density on Target Binding and Sensor Performance

The aptamer’s surface packing density proved to have a significant effect on target binding [167]. The optimal aptamer surface density is also influenced by the size and other properties of the target [167]. Early reports from Plaxco’s group explored the influence of aptamer surface density on the performance of electrochemical aptasensors for small and large molecules targets such as cocaine, and thrombin, respectively [168]. In the case of cocaine sensors, the study revealed a lower sensitivity for densely packed aptamers as a result of the unfavorable interactions between neighboring biomolecules [168]. MacDonald et al. analyzed by SPR and QCM-D the influence of aptamer packing density on the recognition process of a low-molecular weight compound, L-tyrosinamide. The results demonstrated that the affinity for the target was improved when decreasing the surface coverage, as no steric hindrance of the aptamer folding occurred upon analyte recognition. The decrease in K*_D_* when the surface concentration decreased could be justified by SPR results that showed a higher kinetic association rate, whereas the kinetic dissociation rate remained constant, independent of the aptamer surface density [85]. However, very low surface densities, which would provide an unhindered binding, are expected to determine an important decrease in the detection signal and the analytical accuracy would also be low [167]. In consequence, an optimization of the receptor surface density is recommended in order to enable the detection with adequate sensitivity, but at the same time to avoid an intense electrostatic repulsion that would impede an optimal folding of the aptamer [6]. In this context, the employment of diazonium-grafted aromatic groups as anchors for aptamers’ immobilization could provide an efficient control of the surface density [150]. In particular, we recommend the use of protected aryldiazonium salts for electrografting, as they proved their capability for obtaining monolayers with controlled distribution [169,170,171]. A relevant example is the research performed by Leroux et al. on a series of protected phenylacetylene compounds, which demonstrated the possibility of fine-tuning the aryl groups’ surface coverage by simply changing the size of the protecting group from trimethylsilyl to triethylsilyl and tri(isopropyl)silyl [170].

**Figure 4:**
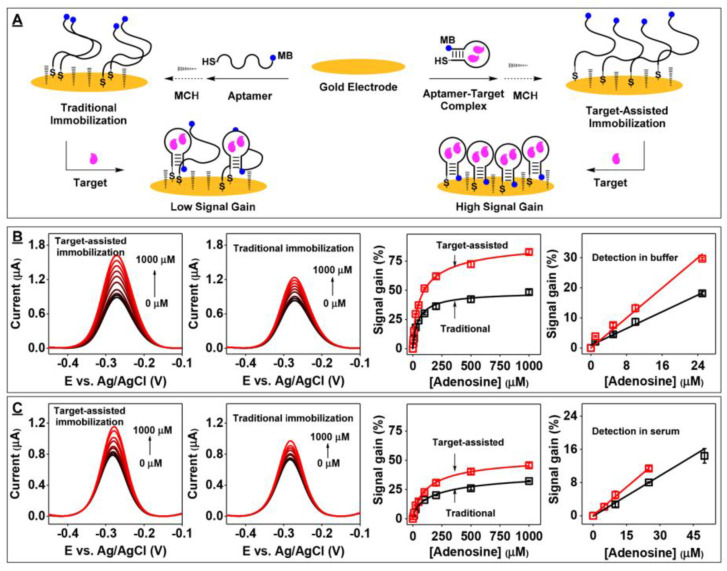
(**A**) The schematic representation of the target-assisted aptamer immobilization procedure for fabrication of an adenosine (ADE) sensor at gold electrodes. The immobilization of the aptamer was performed either through the conventional method (**left**) or by the target-assisted immobilization approach (**right**); SWV measurements in detection and the corresponding calibration curves in (**B**) buffer or (**C**) 50% serum for the aptasensors obtained by target-assisted immobilization (red) or the conventional method (black). Reprinted with permision from ref. [172]. Copyright 2021, American Chemical Society.

Very recently, Xiao’s group reported a novel aptamer immobilization approach, entitled target-assisted aptamer immobilization (Figure 4), that allowed a better control of the immobilized aptamers’ spacing to ensure an optimal target binding, folding, and signal transduction [172]. The study, performed for three different small molecules (adenosine, cocaine, and methylenedioxypyrovalerone) binding aptamers, showed that the immobilization of the aptamers in their target-bound (folded) state onto the electrodes led to a detection platform that provided a higher sensitivity and signal-to-noise ratio than electrochemical sensors fabricated by conventional methods. Another novel feature of the procedure was the use of a low ionic strength buffer instead of conventional high ionic strength conditions, which also significantly improved the performance of the assays [172].

### 6.3. The Influence of the Immobilization Conditions

Recent studies have confirmed that the aptamer surface density alone cannot predict with sufficient certainty the interfacial molecular recognition efficiency, and therefore the influence of other important factors was investigated [172,173].

The studies regarding the type of buffer used for aptamer immobilization, when maintaining the same ionic strength and pH of the medium, showed that the system does not affect the performance of the electrochemical aptasensors [172]. It was also known that high ionic strength buffers led to the increase in the surface concentration of the aptamers, because of the shielding role that the high concentration of cations have on the negatively charged phosphate groups of the oligonucleotides [172]. However, when testing both high and low ionic strength PBS buffer for cocaine aptamer immobilization, at similar surface densities, Xiao and coworkers obtained an improved signal gain for the sensors prepared in low-salt PBS [172]. The authors explained this behavior by a reduced bundling effect for the aptamer molecules at lower ionic strength and, thus, a higher number of bioreceptor molecules available for binding [172]. The effect was previously investigated by Gu et al. which employed high-resolution atomic force microscopy and spatial statistical methods to map and characterize the closely spaced individual receptor probes [173]. Their results demonstrated an increase in oligonucleotide spacing, and therefore a minimization of the biomolecules bundling for a lower ionic strength buffer used during the immobilization procedure [173]. A limited spacing between aptamer receptors hinders the folding and the target binding, and this induces a lower signal in recognition [172]. The conclusion of the study was that the aptamer immobilization should be performed in lower ionic strength conditions, as not even fine-tuning the quantity of aptamer used for immobilization provides the same degree of control [172]. Although one can control at macroscopic level the average spacing between the aptamer molecules confined onto the electrode, still the formation of dense bundles of inactive bioreceptors cannot be prevented for the latter scenario [172].

One important parameter that is not investigated enough but has a significant potential to lead to more predictable sensor preparation procedures is the nature of the cations present in the immobilization media. Nevertheless, there are studies showing that cations promote the aptamers’ folding by reducing the repulsion between the phosphate groups of the nucleic acids, and some ions proved to be more efficient than others. For example, concentrations of Mg^2+^ ions in the mM range can stabilize tertiary structures of aptamers that are only partially stable at higher concentrations of monovalent cations [174]. Chen et al. measured the difference in chain conformation in the presence of Na^+^ and Mg^2+^ cations for two 40-mers oligonucleotides (RNA and ssDNA, respectively) by using a combination of small-angle X-ray scattering and smFRET techniques. For both RNA and ssDNA, it was observed that Mg^2+^ is approximately 20–40 times more efficient for the charge screening than Na^+^ at a similar ionic strength [175].

### 6.4. The Regeneration of the Aptasensors

The robustness of the phosphodiester backbone bestows the aptamers with improved stability [18]. Unlike protein-based bioreceptors such as enzymes or antibodies, aptamers can be reversibly denatured by changing pH, temperature, ionic strength, or in the presence of various reagents, being able to recover their functionality in the original binding conditions [18,22]. Moreover, aptamers are generally able to undergo multiple cycles of denaturation/regeneration [166]. These features make aptasensors amenable to full regeneration, and numerous procedures to reverse the aptamer’s conformation achieved upon target recognition have been reported. Ideally, the regeneration solution would only disrupt the aptamer’s interaction with the target analyte, without affecting its structure. In general, the regeneration procedure consists in an initial and final step of rising with buffer, and, in between the sensor, undergoes the actual treatment proposed for regeneration. The most common strategies include simply rinsing with water, or with concentrated saline, acidic or basic solutions, the use of chaotropic agents such as urea or guanidinium hydrochloride, of chelating agents such as EDTA, changes of temperature, pH or the use of detergents [146,166]. However, a combination of two or more of the mentioned regenerating strategies may be also employed. Other more complex solutions involve interactions with other biomolecules [146].

## 7. Electrochemical Aptasensors for Small Molecules based on Target Binding-Induced Aptamer Conformation Change

It was shown that the conformational change of the aptamers upon binding their matching targets represents a favorable mechanism that can be exploited in the sensors’ design. Most commonly, electrochemical sensors functioning on such mechanisms employ aptamers covalently labeled with electroactive groups. The interaction of the aptamer with its target induces a change in the distance of the label from the electrode, and consequently in the redox signal [176]. Two principles are encountered: the “signal-on” strategy, when the folding of the aptamer in the presence of the analyte favors the approach of the redox label to the electrode, thus increasing the response signal, and the opposite case, the “signal-off” strategy, where the distance between the label and the electrode increases after the target binding, and the signal decreases [168,177,178]. Numerous electrochemical aptasensors functioning on these two approaches have been reported for the detection of small-molecule analytes such as adenosine [115,122,123], cocaine [125,126], different toxins [179], antibiotics [180], etc.

**Figure 5:**
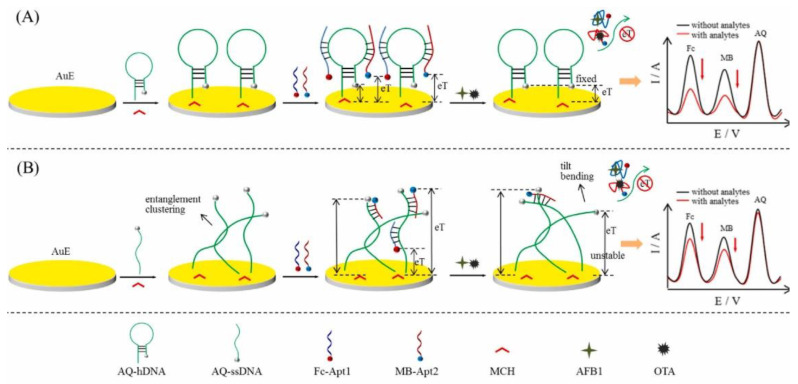
Schematic representation of the working principle of the dual-ratiometric electrochemical aptasensor for simultaneous determination of AFB1 and OTA. The complementary probe employed for the assembly of the AFB1- and OTA-labeled aptamers at the sensor surface had a hairpin (**A**) or a linear (**B**) structure. Reproduced with permission from ref. [184]. Copyright 2022, Elsevier.

The ratiometric approach, a strategy that was a broadly used in fluorescence assays [181], became more frequently used in the last years in the fabrication of the electrochemical aptasensors due to its superior reliability, and reproducibility [182]. The method employs also redox-labeled aptamers and exploits their conformational change following target recognition. The ratiometric electrochemical sensors possess an internal reference that improves the detection accuracy by correcting uncontrollable influences caused by environmental and other factors, and therefore provide two or more electrochemical signals [183]. The analyte is quantified by the ratio of the response signals and the reference signal [182,183,184,185]. A very recent example is a dual-ratiometric electrochemical aptasensing platform developed by Zhu et al. that was applied for the simultaneous detection of two mycotoxins, aflatoxin B1 (AFB1) and Ochratoxin A (OTA) [184]. The aptamers selected for each target were labeled with different redox tags: ferrocene (Fc) for AFB1 aptamer and methylene blue (MB) for OTA aptamer. A complementary DNA sequence (cDNA), that was previously attached by self-assembly at the substrate gold electrode, provided separate and specific hybridization sites to assemble the two labeled aptamers, and to construct the detection interface. The cDNA, labeled by a third redox molecule-anthraquinone (AQ), had a hairpin conformation. The redox signal of AQ, Fc and MB groups was further used to simultaneously produce a reference signal (from AQ) and the two other signals of the redox labels. The targets’ presence determined conformation changes for AFB1 and OTA aptamers that induced their release from cDNA, and consequently a decrease in the Fc and MB redox signals. The relative decrease in these signals with reference to the AQ signal was used to quantify the concentration of the analytes. The working principle of the presented ratiometric aptasensor is depicted in Figure 5. Moreover, the authors emphasized the importance of using a hairpin structure for the cDNA probe, instead of a linear one, for improving the recognition efficiency and selectivity of the assay. The aptasensor exhibited detection ranges of 10–3000 pg mL^−1^ for AFB1 and 30–10,000 pg mL^−1^ for OTA, respectively, and no cross-reactivity. The LODs were 0.0043 ng mL^−1^ for AFFB1 and 0.0133 ng mL^−1^ for OTA, and the sensors could be successfully applied for real sample analysis, such as corn and wheat [184].

A short review of several electrochemical aptasensors based on target-induced aptamer conformation change (developed in the last 3 years), in terms of design (aptamer sequence, affinity, immobilization strategy, detection principle) and the obtained analytical performance is included in Table 1.

## 8. Conclusions

This review provides a short overview of the most important criteria that need to be taken into account when designing electrochemical aptasensors for small-molecule analytes. Because the screening process for small molecule aptamers can be very challenging, there is a constant need of new selection techniques, but the next steps towards further improvements must be built on a good knowledge of the current aptamer selection methodology, on the elucidation of the thermodynamic and structural aspects regarding the aptamer-target interaction, and on consistent standards for evaluating the affinity of aptamers [29]. As the main focus of this review are those approaches that employ aptamer-conformational changes for detecting binding events, a large part of our discussion is dedicated to aptamers with a built-in structure-switching functionality and the strategies commonly employed for modulating their switching thermodynamics [44,99,100]. The recognition ability for these types of receptors is to a large extent reliant on their conformation, therefore a deep knowledge of the influence of the immobilization at the transducer surface and of the detection conditions on the aptamers’ conformation will help in designing effective, reliable and stable biosensors. In this context, several important aspects related to the fabrication of aptamer-modified electrodes suitable for electrochemical sensing, such as the selection of an appropriate immobilization strategy and conditions, are also discussed. Despite the considerable progress made in this area, there are still significant challenges related to the stability, reproducibility, and the reusability of aptasensing platforms. Although in several cases reversing the aptamer’s conformation after target binding could be accomplished by a simple rinse with water, often the regeneration process is more complex, limiting the sensor’s reusability. Furthermore, since many existing aptasensors were developed with simple models such as spiked samples in standard buffers, their robustness must be demonstrated in more complex matrices, such as real environmental samples, beverages and food samples, or biological fluids. We believe that a deep understanding of the factors influencing the screening of small-molecule aptamers and of the main principles for designing efficient detection platforms are fundamental for the future development of aptasensors towards real-world applications. Moreover, further simplification of their fabrication procedures and operating principle are important steps towards the aptasensors’ commercial success. Despite the remaining challenges, by validation with real samples and strategic marketing, a higher impact of the aptasensors is expected [194]. A significant breakthrough in the aptasensing field has already been delivered by the integration of various nanomaterials, both in terms of transduction method optimization and signal amplification. Therefore, we foresee that nanotechnology-based aptasensing platforms will be extensively used in the near future to perform highly sensitive, selective, time and cost-efficient detection of small molecule analytes.

## Figures and Tables

**Table 1:** Electrochemical aptasensors for several small molecules based on conformational change developed in the last 3 years.

Analyte	Apt. Seq.; Tags	K*_D_* Original Seq.; Selection Ref.	Apt. Truncation/Engineering	Detection Method/Immobilization Substrate	Conf. Change/Motif; Regeneration (Y/N)	LOD/Dynamic Range/Real Sample	Ref.
Cortisol	(MB) 5′-NH2 ATGGGCAATGCGGGGTGGAGAATGGTTGCCGCACTTCGGC-SH-3′	6.9 ± 2.8 μM; [186]	constant primer binding seq. deleted (Computational modelling)	SWV; Au	Conf. switch/not mentioned; Y—not mentioned	0.05 ng mL^−1^/0.05–100 ng mL^−1^/serum	[187]
Cortisol	5′-NH2 GGAATGGATCCACATCCATG GATGGGCAATGCGGGGTGGAGAATGGTTGCCGCA CTTCGGCTTCACTGCAGA CTTGACGAAGCTT	6.9 ± 2.8 μM; [186]	Not modified	Label free/CV—capacitance; PDMS@CNC/CNT	Conf. switch/not mentioned/N	1.80 ng mL^−1^/2.50–35 ng mL^−1^/human sweat	[188]
Cortisol	5′-SH (CH2)6-T TTT AGC AG C ACA GAG GTC AGA TGC AAA CCA CAC CTG AGT GGT TAG CGT ATG TCA TTT ACG GC TTT T-3′ (MB) [186]	6.9 ± 2.8 μM; [186]	Tetrathymine spacer attached	DPV; CA/Au/BSA/AuNW	Conf. switch/not mentioned; Y—PBS and 1 M NaCl at pH 4.5 for 15 min	0.51 and 0.68 nM in spiked buffer and undiluted serum samples resp.	[189]
AFB1	5′-SH CACGTGTTGTCTCTCTGTGTCTCGTG-3′ (MB)	49 nM [190]	Not modified	Signal-on; SWV; Au	Conf. switch/stem-loop; Y—rinsing with deionized water	6 pM; 8 pM–25 nM and 25 nM–3 μM; wine, milk and corn flour samples	[30]
AFB1	(COOH) 5′-GATCGGGTCTGGGTGGCGTAAAGGGAGCAT CGGACA-3′(MB)	Not mentioned	Not mentioned	Signal-on; DPV; SPCE/PT3C/HMDA	Conf. switch G-quadruplex; N	1.60 ng L^−1^; 2.50–30 ng L^−1^; coffee samples	[179]
Dexamethasone	(HOC6-S-S-C6) 5′-ACA CGA CGA GGG ACG AGG AGT ACT TGC CAA CGA TAA CGT CGT TGG ATC TGT CTG TGC CC-3′	18.35 nM [191]	spacer attached	EIS ([Fe(CN)_6_]^4−/3−^ redox couple in solution); Au	Conf. switch/not mentioned/N	2.12 nM; 2.50–100 nM; water samples.	[191]
di(2-ethylhexyl) Phthalate	5′-HS-ATG CGG ATC CCG CGC GAC CAA CGG AAG CGC GGC ACC ACA ACG GTG GCG CGA AGC TTG CGC-MB-3′	213 nM [192]	Not modified	Signal-off; DPV; AuFs/NCs/SPCE	Conf. switch/not mentioned/N	0.023 pg mL^−1^; 0.50–10^6^ pg mL^−1^	[193]
Sulfametazine	5′-NH 2-TTA GCT TAT GCG TTG GCC GGG ATA AGG ATC CAG CCG TTG TAG ATT TGC GTT CTA ACT CTC-3′ ([Ru(NH_3_)_6_]^3+^ electrostatic indicator)	Not mentioned	Not mentioned	Signal-on; DPV; PEI/CQDs-WS2/GCE	Conf. switch/not mentioned/N	4 pM; 10 pM–1 µM; food and environmental samples	[180]
AFB1 + OTA	AFB1: 5′-Fc-GTTGGGCACGTGTTGTCTCTCTGTGTCTCGTGCCCTTCGCTAGGCCC-3′ OTA: 5′-GATCGGGTGTGGGTG GCGTAAAGGGAGCATCGACAMB-3′	Not mentioned	Not mentioned	Signal-off; ACV; AQ-hDNA/Au	Conf. switch/not mentioned/N	AFB1: 0.0043 ng mL^−1^; OTA: 0.0133 ng mL^−1^; AFB1: 10–3000 pg mL^−1^; OTA: 30–10,000 pg mL^−1^; corn and wheat	[184]
ATP	5′-Fc-(CH2)6-ACC TGG GGG AGT ATT GCG GAG GAA GGT-(CH2)6-SH-3′	6 ± 3 μM [109]	spacer attached	Signal-on; ACV; Au MEA chip	Conf. switch G-quadruplex; N	0.30 nM; 0.50–50 nM; diluted human serum and artificial cerebrospinal fluid	[123]

Apt—aptamer; seq.—sequence; conf.—conformation; SWV—square wave voltammetry; CV—cyclic voltammetry; PDMS—polydimethylsiloxane; CNC/CNT—multiwalled carbon nanotube/cellulose nanocrystal; MB—methylene blue; DPV—differential pulse voltammetry; CA—chronoamperometry; AFB1—aflatoxin B1; OTA—ochratoxin A; AuNW—gold nanowire; SPCE—screen printed electrode; PT3C—polythiophene-3-carboxylic acid; HMDA—hexamethylenediamine; EIS—electrochemical impedance spectroscopy; AuFs—gold nanoflowers; NCs—hybrid nanocomposites of molybdenum disulfide nanosheets (MoS_2_NSs), graphene nanoplatelets (GNPs) and chitosan (CHT); PEI—polyethyleneimine; CQDs-WS2—carbon quantum dots-tungsten disulfide nanocomposite; GCE—glassy carbon electrode; ACV—Alternating current voltammetry; AQ-hDNA—antraquinone labeled hairpin DNA; MEA—multielectrode array.

## Data Availability

Not applicable.

## References

[1] Raicopol M., Pilan L. (2021). The Role of Aryldiazonium Chemistry in Designing Electrochemical Aptasensors for the Detection of Food Contaminants. Materials.

[2] Riu J., Giussani B. (2020). Electrochemical biosensors for the detection of pathogenic bacteria in food. TrAC Trends Anal. Chem..

[3] Zara L., Achilli S., Chovelon B., Fiore E., Toulmé J.-J., Peyrin E., Ravelet C. (2021). Anti-pesticide DNA aptamers fail to recognize their targets with asserted micromolar dissociation constants. Anal. Chim. Acta.

[4] Yu H., Alkhamis O., Canoura J., Liu Y., Xiao Y. (2021). Advances and Challenges in Small-Molecule DNA Aptamer Isolation, Characterization, and Sensor Development. Angew. Chem. Int. Ed..

[5] Wang Z.-J., Chen E.-N., Yang G., Zhao X.-Y., Qu F. (2020). Research Advances of Aptamers Selection for Small Molecule Targets. Chin. J. Anal. Chem..

[6] Prante M., Segal E., Scheper T., Bahnemann J., Walter J. (2020). Aptasensors for Point-Of-Care Detection of Small Molecules. Biosensors.

[7] Yang Y., Yan W., Guo Y., Wang X., Zhang F., Yu L., Guo C., Fang G. (2020). Sensitive and selective electrochemical aptasensor via diazonium-coupling reaction for label-free determination of oxytetracycline in milk samples. Sens. Actuators Rep..

[8] Zhao L., Qi X., Yan X., Huang Y., Liang X., Zhang L., Wang S., Tan W. (2019). Engineering Aptamer with Enhanced Affinity by Triple Helix-Based Terminal Fixation. J. Am. Chem. Soc..

[9] Dunn M.R., Jimenez R.M., Chaput J.C. (2017). Analysis of aptamer discovery and technology. Nat. Rev. Chem..

[10] Walter J.-G., Heilkenbrinker A., Austerjost J., Timur S., Stahl F., Schepe T. (2012). Aptasensors for Small Molecule Detection. Z. Für Nat. B.

[11] Ganguly A., Lin K.C., Muthukumar S., Prasad S. (2021). Autonomous, Real-Time Monitoring Electrochemical Aptasensor for Circadian Tracking of Cortisol Hormone in Sub-microliter Volumes of Passively Eluted Human Sweat. ACS Sens..

[12] Rapini R., Marrazza G. (2017). Electrochemical aptasensors for contaminants detection in food and environment: Recent advances. Bioelectrochemistry.

[13] Tuerk C., Gold L. (1990). Systematic Evolution of Ligands by Exponential Enrichment: RNA Ligands to Bacteriophage T4 DNA Polymerase. Science.

[14] Ellington A.D., Szostak J.W. (1990). In vitro selection of RNA molecules that bind specific ligands. Nature.

[15] Zhang Z., Oni O., Liu J. (2017). New insights into a classic aptamer: Binding sites, cooperativity and more sensitive adenosine detection. Nucleic Acids Res..

[16] Sakamoto T., Ennifar E., Nakamura Y. (2018). Thermodynamic study of aptamers binding to their target proteins. Biochimie.

[17] Eaton B.E., Gold L., Zichi D.A. (1995). Let’s get specific: The relationship between specificity and affinity. Chem. Biol..

[18] McKeague M., DeRosa M.C. (2012). Challenges and Opportunities for Small Molecule Aptamer Development. J. Nucleic Acids.

[19] Evtugyn G., Porfireva A., Shamagsumova R., Hianik T. (2020). Advances in Electrochemical Aptasensors Based on Carbon Nanomaterials. Chemosensors.

[20] Prieto-Simon B., Campas M., Marty J.-L. (2008). Biomolecule Immobilization in Biosensor Development: Tailored Strategies Based on Affinity Interactions. Protein Pept. Lett..

[21] Qi X., Yan X., Zhao Y., Li L., Wang S. (2020). Highly sensitive and specific detection of small molecules using advanced aptasensors based on split aptamers: A review. TrAC Trends Anal. Chem..

[22] Ștefan G., Hosu O., De Wael K., Lobo-Castañón M.J., Cristea C. (2021). Aptamers in biomedicine: Selection strategies and recent advances. Electrochim. Acta.

[23] Pfeiffer F., Mayer G. (2016). Selection and Biosensor Application of Aptamers for Small Molecules. Front. Chem..

[24] Sharma T.K., Bruno J.G., Dhiman A. (2017). ABCs of DNA aptamer and related assay development. Biotechnol. Adv..

[25] McKeague M., De Girolamo A., Valenzano S., Pascale M., Ruscito A., Velu R., Frost N.R., Hill K., Smith M., McConnell E.M. (2015). Comprehensive Analytical Comparison of Strategies Used for Small Molecule Aptamer Evaluation. Anal. Chem..

[26] Jenison R.D., Gill S.C., Pardi A., Polisky B. (1994). High-Resolution Molecular Discrimination by RNA. Science.

[27] Fickert H., Fransson I.G., Hahn U. (2006). In Vitro Selection of Target-Binding Oligonucleotides Aptamers to Small Molecules. The Aptamer Handbook.

[28] Yang Y., Kochoyan M., Burgstaller P., Westhof E., Famulok M. (1996). Structural Basis of Ligand Discrimination by Two Related RNA Aptamers Resolved by NMR Spectroscopy. Science.

[29] Sester C., McCone J.A., Sen A., Vorster J., Harvey J.E., Hodgkiss J.M. (2022). Unraveling the binding mode of a methamphetamine aptamer: A spectroscopic and calorimetric study. Biophys. J..

[30] Wang C., Li Y., Zhao Q. (2019). A signal-on electrochemical aptasensor for rapid detection of aflatoxin B1 based on competition with complementary DNA. Biosens. Bioelectron..

[31] Reinstein O., Yoo M., Han C., Palmo T., Beckham S.A., Wilce M.C.J., Johnson P.E. (2013). Quinine Binding by the Cocaine-Binding Aptamer. Thermodynamic and Hydrodynamic Analysis of High-Affinity Binding of an Off-Target Ligand. Biochemistry.

[32] Melaine F., Coilhac C., Roupioz Y., Buhot A. (2016). A nanoparticle-based thermo-dynamic aptasensor for small molecule detection. Nanoscale.

[33] Farjami E., Campos R., Nielsen J.S., Gothelf K.V., Kjems J., Ferapontova E.E. (2013). RNA Aptamer-Based Electrochemical Biosensor for Selective and Label-Free Analysis of Dopamine. Anal. Chem..

[34] Labuda J., Brett A.M.O., Evtugyn G., Fojta M., Mascini M., Ozsoz M., Palchetti I., Paleček E., Wang J. (2010). Electrochemical nucleic acid-based biosensors: Concepts, terms, and methodology (IUPAC Technical Report). Pure Appl. Chem..

[35] Cheng A.K., Sen D., Yu H.-Z. (2009). Design and testing of aptamer-based electrochemical biosensors for proteins and small molecules. Bioelectrochemistry.

[36] Wang C., Zhao Q. (2020). A reagentless electrochemical sensor for aflatoxin B1 with sensitive signal-on responses using aptamer with methylene blue label at specific internal thymine. Biosens. Bioelectron..

[37] Zhou L., Wang J., Li D., Li Y. (2014). An electrochemical aptasensor based on gold nanoparticles dotted graphene modified glassy carbon electrode for label-free detection of bisphenol A in milk samples. Food Chem..

[38] Liu R., Yang Z., Guo Q., Zhao J., Ma J., Kang Q., Tang Y., Xue Y., Lou X., He M. (2015). Signaling-Probe Displacement Electrochemical Aptamer-based Sensor (SD-EAB) for Detection of Nanomolar Kanamycin A. Electrochim. Acta.

[39] Liu S., Wang Y., Xu W., Leng X., Wang H., Guo Y., Huang J. (2017). A novel sandwich-type electrochemical aptasensor based on GR-3D Au and aptamer-AuNPs-HRP for sensitive detection of oxytetracycline. Biosens. Bioelectron..

[40] Li F., Yu Z., Han X., Lai R.Y. (2019). Electrochemical aptamer-based sensors for food and water analysis: A review. Anal. Chim. Acta.

[41] Ozer A., Pagano J.M., Lis J.T. (2014). New Technologies Provide Quantum Changes in the Scale, Speed, and Success of SELEX Methods and Aptamer Characterization. Mol. Ther. Nucleic Acids.

[42] Lyu C., Khan I.M., Wang Z. (2021). Capture-SELEX for aptamer selection: A short review. Talanta.

[43] Wang T., Chen C., Larcher L.M., Barrero R.A., Veedu R.N. (2019). Three decades of nucleic acid aptamer technologies: Lessons learned, progress and opportunities on aptamer development. Biotechnol. Adv..

[44] Alkhamis O., Canoura J., Yu H., Liu Y., Xiao Y. (2019). Innovative engineering and sensing strategies for aptamer-based small-molecule detection. TrAC Trends Anal. Chem..

[45] Wilson C., Nix J., Szostak J. (1998). Functional Requirements for Specific Ligand Recognition by a Biotin-Binding RNA Pseudoknot. Biochemistry.

[46] Mendonsa S.D., Bowser M.T. (2005). In Vitro Selection of Aptamers with Affinity for Neuropeptide Y Using Capillary Electrophoresis. J. Am. Chem. Soc..

[47] Nutiu R., Li Y. (2005). In Vitro Selection of Structure-Switching Signaling Aptamers. Angew. Chem. Int. Ed..

[48] Stoltenburg R., Nikolaus N., Strehlitz B. (2012). Capture-SELEX: Selection of DNA Aptamers for Aminoglycoside Antibiotics. J. Anal. Methods Chem..

[49] Eruscito A., DeRosa M.C. (2016). Small-Molecule Binding Aptamers: Selection Strategies, Characterization, and Applications. Front. Chem..

[50] Park J.-W., Tatavarty R., Kim D.W., Jung H.-T., Gu M.B. (2011). Immobilization-free screening of aptamers assisted by graphene oxide. Chem. Commun..

[51] Zhuo Z., Yu Y., Wang M., Li J., Zhang Z., Liu J., Wu X., Lu A., Zhang G., Zhang B. (2017). Recent Advances in SELEX Technology and Aptamer Applications in Biomedicine. Int. J. Mol. Sci..

[52] Derbyshire N., White S.J., Bunka D.H.J., Song L., Stead S., Tarbin J., Sharman M., Zhou D., Stockley P.G. (2012). Toggled RNA Aptamers Against Aminoglycosides Allowing Facile Detection of Antibiotics Using Gold Nanoparticle Assays. Anal. Chem..

[53] Yang W., Yu H., Alkhamis O., Liu Y., Canoura J., Fu F., Xiao Y. (2019). In vitro isolation of class-specific oligonucleotide-based small-molecule receptors. Nucleic Acids Res..

[54] Yu H., Luo Y., Alkhamis O., Canoura J., Yu B., Xiao Y. (2021). Isolation of Natural DNA Aptamers for Challenging Small-Molecule Targets, Cannabinoids. Anal. Chem..

[55] Ruscito A., McConnell E.M., Koudrina A., Velu R., Mattice C., Hunt V., McKeague M., DeRosa M.C. (2017). In Vitro Selection and Characterization of DNA Aptamers to a Small Molecule Target. Curr. Protoc. Chem. Biol..

[56] Famulok M., Mayer G. (2014). Aptamers and SELEX in Chemistry & Biology. Chem. Biol..

[57] Komarova N., Barkova D., Kuznetsov A. (2020). Implementation of High-Throughput Sequencing (HTS) in Aptamer Selection Technology. Int. J. Mol. Sci..

[58] Phopin K., Tantimongcolwat T. (2020). Pesticide Aptasensors—State of the Art and Perspectives. Sensors.

[59] Rozenblum G.T., Pollitzer I.G., Radrizzani M. (2019). Challenges in Electrochemical Aptasensors and Current Sensing Architectures Using Flat Gold Surfaces. Chemosensors.

[60] Kastritis P., Bonvin A.M.J.J. (2013). On the binding affinity of macromolecular interactions: Daring to ask why proteins interact. J. R. Soc. Interface.

[61] Kalra P., Dhiman A., Cho W.C., Bruno J.G., Sharma T.K. (2018). Simple Methods and Rational Design for Enhancing Aptamer Sensitivity and Specificity. Front. Mol. Biosci..

[62] Thevendran R., Navien T.N., Meng X., Wen K., Lin Q., Sarah S., Tang T.-H., Citartan M. (2020). Mathematical approaches in estimating aptamer-target binding affinity. Anal. Biochem..

[63] Sassolas A., Blum L.J., Leca-Bouvier B.D. (2009). Electrochemical Aptasensors. Electroanalysis.

[64] Mattarozzi M., Toma L., Bertucci A., Giannetto M., Careri M. (2022). Aptamer-based assays: Strategies in the use of aptamers conjugated to magnetic micro- and nanobeads as recognition elements in food control. Anal. Bioanal. Chem..

[65] Svobodova M., Skouridou V., Botero M.L., Jauset-Rubio M., Schubert T., Bashammakh A.S., El-Shahawi M.S., Alyoubi A.O., O’Sullivan C.K. (2017). The characterization and validation of 17β-estradiol binding aptamers. J. Steroid Biochem. Mol. Biol..

[66] Chang A.L., McKeague M., Liang J.C., Smolke C.D. (2014). Kinetic and Equilibrium Binding Characterization of Aptamers to Small Molecules using a Label-Free, Sensitive, and Scalable Platform. Anal. Chem..

[67] Hasegawa H., Savory N., Abe K., Ikebukuro K. (2016). Methods for Improving Aptamer Binding Affinity. Molecules.

[68] de Girolamo A., McKeague M., Pascale M., Cortese M., DeRosa M.C. (2018). Immobilization of Aptamers on Substrates. Aptamers for Analytical Applications.

[69] Azri F.A., Selamat J., Sukor R., Yusof N.A., Raston N.H.A., Eissa S., Zourob M., Chinnappan R. (2021). Determination of minimal sequence for zearalenone aptamer by computational docking and application on an indirect competitive electrochemical aptasensor. Anal. Bioanal. Chem..

[70] Hu J., Easley C. (2011). A simple and rapid approach for measurement of dissociation constants of DNA aptamers against proteins and small molecules via automated microchip electrophoresis. Analyst.

[71] Cai S., Yan J., Xiong H., Liu Y., Peng D., Liu Z. (2018). Investigations on the interface of nucleic acid aptamers and binding targets. Analyst.

[72] Slavkovic S., Altunisik M., Reinstein O., Johnson P.E. (2015). Structure–affinity relationship of the cocaine-binding aptamer with quinine derivatives. Bioorganic Med. Chem..

[73] Slavkovic S., Johnson P.E. (2018). PROTOCOL/METHOD Isothermal Titration Calorimetry Studies of Aptamer-Small Molecule Inter-Actions: Practicalities and Pitfalls. Aptamers.

[74] Neves M.A., Reinstein O., Saad M., Johnson P.E. (2010). Defining the secondary structural requirements of a cocaine-binding aptamer by a thermodynamic and mutation study. Biophys. Chem..

[75] Challier L., Miranda-Castro R., Barbe B., Fave C., Limoges B., Peyrin E., Ravelet C., Fiore E., Labbé P., Coche-Guérente L. (2016). Multianalytical Study of the Binding between a Small Chiral Molecule and a DNA Aptamer: Evidence for Asymmetric Steric Effect upon 3′- versus 5′-End Sequence Modification. Anal. Chem..

[76] Baaske P., Wienken C.J., Reineck P., Duhr S., Braun D. (2010). Optical Thermophoresis for Quantifying the Buffer Dependence of Aptamer Binding. Angew. Chem. Int. Ed..

[77] Rangel A.E., Chen Z., Ayele T., Heemstra J.M. (2018). In vitro selection of an XNA aptamer capable of small-molecule recognition. Nucleic Acids Res..

[78] Thevendran R., Citartan M. (2022). Assays to Estimate the Binding Affinity of Aptamers. Talanta.

[79] Drabovich A.P., Berezovski M., Okhonin V., Krylov S.N. (2006). Selection of Smart Aptamers by Methods of Kinetic Capillary Electrophoresis. Anal. Chem..

[80] Bock L.C., Griffin L.C., Latham J.A., Vermaas E.H., Toole J.J. (1992). Selection of single-stranded DNA molecules that bind and inhibit human thrombin. Nature.

[81] Jhaveri S.D., Ellington A.D. (2000). In Vitro Selection of RNA Aptamers to a Protein Target by Filter Immobilization. Curr. Protoc. Mol. Biol..

[82] Donaldson G.P., Roelofs K.G., Luo Y., Sintim H.O., Lee V.T. (2012). A rapid assay for affinity and kinetics of molecular interactions with nucleic acids. Nucleic Acids Res..

[83] Okazawa A., Maeda H., Fukusaki E., Katakura Y., Kobayashi A. (2000). In vitro selection of hematoporphyrin binding DNA aptamers. Bioorganic Med. Chem. Lett..

[84] Kwon M., Chun S.M., Jeong S., Yu J. (2001). In vitro selection of RNA against kanamycin B. Mol. Cells.

[85] MacDonald H., Bonnet H., Van der Heyden A., Defrancq E., Spinelli N., Coche-Guérente L., Dejeu J. (2019). Influence of Aptamer Surface Coverage on Small Target Recognition: A SPR and QCM-D Comparative Study. J. Phys. Chem. C.

[86] Win M.N., Klein J.S., Smolke C.D. (2006). Codeine-binding RNA aptamers and rapid determination of their binding constants using a direct coupling surface plasmon resonance assay. Nucleic Acids Res..

[87] Özalp V.C. (2011). Acoustic quantification of ATP using a quartz crystal microbalance with dissipation. Analyst.

[88] Durand G., Lisi S., Ravelet C., Dausse E., Peyrin E., Toulmé J.-J. (2014). Riboswitches Based on Kissing Complexes for the Detection of Small Ligands. Angew. Chem. Int. Ed..

[89] Hermann T., Patel D.J. (2000). Adaptive Recognition by Nucleic Acid Aptamers. Science.

[90] Ming T., Wang Y., Luo J., Liu J., Sun S., Xing Y., Xiao G., Jin H., Cai X. (2019). Folding Paper-Based Aptasensor Platform Coated with Novel Nanoassemblies for Instant and Highly Sensitive Detection of 17β-Estradiol. ACS Sens..

[91] Zhang J., Chen J., Zhang X., Zeng Z., Chen M., Wang S. (2012). An electrochemical biosensor based on hairpin-DNA aptamer probe and restriction endonuclease for ochratoxin A detection. Electrochem. Commun..

[92] Chiorcea-Paquim A.-M., Oliveira-Brett A.M. (2016). Guanine Quadruplex Electrochemical Aptasensors. Chemosensors.

[93] Malecka K., Ferapontova E.E. (2021). Femtomolar Detection of Thrombin in Serum and Cerebrospinal Fluid via Direct Electrocatalysis of Oxygen Reduction by the Covalent G4-Hemin-Aptamer Complex. ACS Appl. Mater. Interfaces.

[94] Goux E., Lisi S., Ravelet C., Durand G., Fiore E., Dausse E., Toulmé J.-J., Peyrin E. (2015). An improved design of the kissing complex-based aptasensor for the detection of adenosine. Anal. Bioanal. Chem..

[95] Dejeu J., Van der Heyden A., Spinelli N., Defrancq E., Coche-Guérente L. (2021). Recent progress in the design of G-quadruplex–based electrochemical aptasensors. Curr. Opin. Electrochem..

[96] Le T.T., Chumphukam O., Cass A.E.G. (2014). Determination of minimal sequence for binding of an aptamer. A comparison of truncation and hybridization inhibition methods. RSC Adv..

[97] Bernat V., Disney M.D. (2015). RNA Structures as Mediators of Neurological Diseases and as Drug Targets. Neuron.

[98] Wang Z., Yu H., Canoura J., Liu Y., Alkhamis O., Fu F., Xiao Y. (2018). Introducing structure-switching functionality into small-molecule-binding aptamers via nuclease-directed truncation. Nucleic Acids Res..

[99] Feagin T.A., Maganzini N., Soh H.T. (2018). Strategies for Creating Structure-Switching Aptamers. ACS Sens..

[100] Vallée-Bélisle A., Ricci F., Plaxco K.W. (2009). Thermodynamic basis for the optimization of binding-induced biomolecular switches and structure-switching biosensors. Proc. Natl. Acad. Sci. USA.

[101] Fan C., Plaxco K.W., Heeger A.J. (2003). Electrochemical interrogation of conformational changes as a reagentless method for the sequence-specific detection of DNA. Proc. Natl. Acad. Sci. USA.

[102] Lubin A.A., Plaxco K.W. (2010). Folding-Based Electrochemical Biosensors: The Case for Responsive Nucleic Acid Architectures. Acc. Chem. Res..

[103] Tyagi S., Kramer F.R. (1996). Molecular Beacons: Probes that Fluoresce upon Hybridization. Nat. Biotechnol..

[104] Moutsiopoulou A., Broyles D., Dikici E., Daunert S., Deo S.K. (2019). Molecular Aptamer Beacons and Their Applications in Sensing, Imaging, and Diagnostics. Small.

[105] Han K., Liang Z., Zhou N. (2010). Design Strategies for Aptamer-Based Biosensors. Sensors.

[106] Porchetta A., Vallée-Bélisle A., Plaxco K.W., Ricci F. (2012). Using Distal-Site Mutations and Allosteric Inhibition To Tune, Extend, and Narrow the Useful Dynamic Range of Aptamer-Based Sensors. J. Am. Chem. Soc..

[107] Kang D., Vallée-Bélisle A., Porchetta A., Plaxco K.W., Ricci F. (2012). Re-engineering Electrochemical Biosensors To Narrow or Extend Their Useful Dynamic Range. Angew. Chem. Int. Ed..

[108] Armstrong R.E., Strouse G.F. (2014). Rationally Manipulating Aptamer Binding Affinities in a Stem-Loop Molecular Beacon. Bioconjugate Chem..

[109] Huizenga D.E., Szostak J.W. (1995). A DNA Aptamer That Binds Adenosine and ATP. Biochemistry.

[110] Mitteaux J., Lejault P., Wojciechowski F., Joubert A., Boudon J., Desbois N., Gros C.P., Hudson R.H.E., Boulé J.-B., Granzhan A. (2021). Identifying G-Quadruplex-DNA-Disrupting Small Molecules. J. Am. Chem. Soc..

[111] Rhodes D., Lipps H.J. (2015). Survey and Summary G-quadruplexes and their regulatory roles in biology. Nucleic Acids Res..

[112] Spiegel J., Adhikari S., Balasubramanian S. (2020). The Structure and Function of DNA G-Quadruplexes. Trends Chem..

[113] Awadasseid A., Ma X., Wu Y., Zhang W. (2021). G-quadruplex stabilization via small-molecules as a potential anti-cancer strategy. Biomed. Pharmacother..

[114] Ma D.-L., Wu C., Dong Z.-Z., Tam W.-S., Wong S.-W., Yang C., Li G., Leung C.-H. (2017). The Development of G-Quadruplex-Based Assays for the Detection of Small Molecules and Toxic Substances. Chem. Asian J..

[115] Yu J., Zhang L., Xu X., Liu S. (2014). Quantitative Detection of Potassium Ions and Adenosine Triphosphate via a Nanochannel-Based Electrochemical Platform Coupled with G-Quadruplex Aptamers. Anal. Chem..

[116] Wu Z.-S., Chen C.-R., Shen G.-L., Yu R.-Q. (2008). Reversible electronic nanoswitch based on DNA G-quadruplex conformation: A platform for single-step, reagentless potassium detection. Biomaterials.

[117] Slavkovic S., Zhu Y., Churcher Z.R., Shoara A.A., Johnson A.E., Johnson P.E. (2020). Thermodynamic analysis of cooperative ligand binding by the ATP-binding DNA aptamer indicates a population-shift binding mechanism. Sci. Rep..

[118] Munzar J.D., Ng A., Corrado M., Juncker D. (2017). Complementary oligonucleotides regulate induced fit ligand binding in duplexed aptamers. Chem. Sci..

[119] Lin C.H., Patei D.J. (1997). Structural basis of DNA folding and recognition in an AMP-DNA aptamer complex: Distinct architectures but common recognition motifs for DNA and RNA aptamers complexed to AMP. Chem. Biol..

[120] Biniuri Y., Luo G.-F., Fadeev M., Wulf V., Willner I. (2019). Redox-Switchable Binding Properties of the ATP–Aptamer. J. Am. Chem. Soc..

[121] Shi P., Zhang Y., Yuanchao Z., Zhang S. (2017). Label-free Electrochemical Detection of ATP Based on Amino-functionalized Metal-organic Framework. Sci. Rep..

[122] Kashefi-Kheyrabadi L., Mehrgardi M.A. (2013). Aptamer-based electrochemical biosensor for detection of adenosine triphosphate using a nanoporous gold platform. Bioelectrochemistry.

[123] Wu C., Offenhäusser A., Mayer D. (2020). A Highly Sensitive Amperometric Aptamer Biosensor for Adenosine Triphosphate Detection on a 64 Channel Gold Multielectrode Array. Phys. Status Solidi (A) Appl. Mater. Sci..

[124] Jia J., Feng J., Chen H.G., Luo H.Q., Li N.B. (2016). A simple electrochemical method for the detection of ATP using target-induced conformational change of dual-hairpin DNA structure. Sens. Actuators B Chem..

[125] Baker B.R., Lai R.Y., Wood M.S., Doctor E.H., Heeger A.J., Plaxco K.W. (2006). An Electronic, Aptamer-Based Small-Molecule Sensor for the Rapid, Label-Free Detection of Cocaine in Adulterated Samples and Biological Fluids. J. Am. Chem. Soc..

[126] Swensen J.S., Xiao Y., Ferguson B.S., Lubin A.A., Lai R.Y., Heeger A.J., Plaxco K.W., Soh H.T. (2009). Continuous, Real-Time Monitoring of Cocaine in Undiluted Blood Serum via a Microfluidic, Electrochemical Aptamer-Based Sensor. J. Am. Chem. Soc..

[127] Zhang J., Xing H., Lu Y. (2018). Translating molecular detections into a simple temperature test using a target-responsive smart thermometer. Chem. Sci..

[128] Stojanovic M.N., de Prada P., Landry D.W. (2001). Aptamer-Based Folding Fluorescent Sensor for Cocaine. J. Am. Chem. Soc..

[129] Neves M.A.D., Reinstein O., Johnson P.E. (2010). Defining a Stem Length-Dependent Binding Mechanism for the Cocaine-Binding Aptamer. A Combined NMR and Calorimetry Study. Biochemistry.

[130] Sachan A., Ilgu M., Kempema A., Kraus G.A., Nilsen-Hamilton M. (2016). Specificity and Ligand Affinities of the Cocaine Aptamer: Impact of Structural Features and Physiological NaCl. Anal. Chem..

[131] Hayashi T., Oshima H., Mashima T., Nagata T., Katahira M., Kinoshita M. (2014). Binding of an RNA aptamer and a partial peptide of a prion protein: Crucial importance of water entropy in molecular recognition. Nucleic Acids Res..

[132] Kypr J., Kejnovska I., Renciuk D., Vorlickova M. (2009). Circular dichroism and conformational polymorphism of DNA. Nucleic Acids Res..

[133] Ranjbar B., Gill P. (2009). Circular Dichroism Techniques: Biomolecular and Nanostructural Analyses- A Review. Chem. Biol. Drug Des..

[134] Rowe A.A., Miller E.A., Plaxco K.W. (2010). Reagentless Measurement of Aminoglycoside Antibiotics in Blood Serum via an Electrochemical, Ribonucleic Acid Aptamer-Based Biosensor. Anal. Chem..

[135] Patel D.J., Suri A.K., Jiang F., Jiang L., Fan P., Kumar R., Nonin S. (1997). Structure, recognition and adaptive binding in RNA aptamer complexes. J. Mol. Biol..

[136] Gao S., Zheng X., Jiao B., Wang L. (2016). Post-SELEX optimization of aptamers. Anal. Bioanal. Chem..

[137] Yue F., Li H., Kong Q., Liu J., Wang G., Li F., Yang Q., Chen W., Guo Y., Sun X. (2022). Selection of broad-spectrum aptamer and its application in fabrication of aptasensor for detection of aminoglycoside antibiotics residues in milk. Sens. Actuators B Chem..

[138] Tao X., He F., Liu X., Zhang F., Wang X., Peng Y., Liu J. (2020). Detection of chloramphenicol with an aptamer-based colorimetric assay: Critical evaluation of specific and unspecific binding of analyte molecules. Mikrochim. Acta.

[139] Tsao S.-M., Lai J.-C., Horng H.-E., Liu T.-C., Hong C.-Y. (2017). Generation of Aptamers from A Primer-Free Randomized ssDNA Library Using Magnetic-Assisted Rapid Aptamer Selection. Sci. Rep..

[140] Mehta J., Van Dorst B., Rouah-Martin E., Herrebout W., Scippo M.-L., Blust R., Robbens J. (2011). In vitro selection and characterization of DNA aptamers recognizing chloramphenicol. J. Biotechnol..

[141] Yadav S.K., Agrawal B., Chandra P., Goyal R.N. (2014). In vitro chloramphenicol detection in a Haemophilus influenza model using an aptamer-polymer based electrochemical biosensor. Biosens. Bioelectron..

[142] Wang L., Liu X., Zhang Q., Zhang C., Liu Y., Tu K., Tu J. (2012). Selection of DNA aptamers that bind to four organophosphorus pesticides. Biotechnol. Lett..

[143] Canoura J., Yu H., Alkhamis O., Roncancio D., Farhana R., Xiao Y. (2021). Accelerating Post-SELEX Aptamer Engineering Using Exonuclease Digestion. J. Am. Chem. Soc..

[144] Niazi J.H., Lee S.J., Kim Y.S., Gu M.B. (2008). ssDNA aptamers that selectively bind oxytetracycline. Bioorganic Med. Chem..

[145] Kwon Y.S., Raston N.H.A., Gu M.B. (2014). An ultra-sensitive colorimetric detection of tetracyclines using the shortest aptamer with highly enhanced affinity. Chem. Commun..

[146] Tello A., Cao R., Marchant M.J., Gomez H. (2016). Conformational Changes of Enzymes and Aptamers Immobilized on Electrodes. Bioconjugate Chem..

[147] Rondinini S., Vertova A., Pilan L. (2003). The Use of Hexaamineruthenium(III) Redox Marker for the Characterization of SAM-Au Amperometric Sensors. Electroanalysis.

[148] Radi A.-E., Sánchez J.L.A., Baldrich E., O’Sullivan C.K. (2006). Reagentless, Reusable, Ultrasensitive Electrochemical Molecular Beacon Aptasensor. J. Am. Chem. Soc..

[149] Pan S., Rothberg L. (2005). Chemical Control of Electrode Functionalization for Detection of DNA Hybridization by Electrochemical Impedance Spectroscopy. Langmuir.

[150] Yáñez-Sedeño P., Campuzano S., Pingarrón J.M. (2018). Integrated Affinity Biosensing Platforms on Screen-Printed Electrodes Electrografted with Diazonium Salts. Sensors.

[151] Hetemi D., Noël V., Pinson J. (2020). Grafting of Diazonium Salts on Surfaces: Application to Biosensors. Biosens..

[152] Chiticaru E.A., Pilan L., Ioniţă M. (2022). Electrochemical Detection Platform Based on RGO Functionalized with Diazonium Salt for DNA Hybridization. Biosensors.

[153] Pilan L. (2021). Tailoring the performance of electrochemical biosensors based on carbon nanomaterials via aryldiazonium electrografting. Bioelectrochemistry.

[154] Raicopol M., Vlsceanu I., Lupulescu I., Brezoiu A.M., Pilan L. (2016). Amperometric Glucose Biosensors Based on Functionalized Electrochemically Reduced Graphene Oxide. UPB Sci. Bull. Ser. B Chem. Mater. Sci..

[155] Bagheryan Z., Raoof J.-B., Golabi M., Turner A.P., Beni V. (2016). Diazonium-based impedimetric aptasensor for the rapid label-free detection of Salmonella typhimurium in food sample. Biosens. Bioelectron..

[156] Cao C., Zhang Y., Jiang C., Qi M., Liu G. (2017). Advances on Aryldiazonium Salt Chemistry Based Interfacial Fabrication for Sensing Applications. ACS Appl. Mater. Interfaces.

[157] Pinson J., Podvorica F. (2005). Attachment of organic layers to conductive or semiconductive surfaces by reduction of diazonium salts. Chem. Soc. Rev..

[158] Raicopol M., Andronescu C., Atasiei R., Hanganu A., Manea A.M., Rau I., Kajzar F., Pilan L. (2015). Synthesis of conducting azopolymers by electrochemical grafting of a diazonium salt at polypyrrole electrodes. Synth. Met..

[159] Raicopol M., Andronescu C., Atasiei R., Hanganu A., Pilan L. (2014). Post-Polymerization Electrochemical Functionalization of a Conducting Polymer: Diazonium Salt Electroreduction at Polypyrrole Electrodes. J. Electrochem. Soc..

[160] Levrie K., Jans K., Vos R., Ardakanian N., Verellen N., Van Hoof C., Lagae L., Stakenborg T. (2016). Multiplexed site-specific electrode functionalization for multitarget biosensors. Bioelectrochemistry.

[161] Jiang C., Silva S.M., Fan S., Wu Y., Alam M.T., Liu G., Gooding J.J. (2017). Aryldiazonium salt derived mixed organic layers: From surface chemistry to their applications. J. Electroanal. Chem..

[162] Hayat A., Sassolas A., Marty J.-L., Radi A.-E. (2013). Highly sensitive ochratoxin A impedimetric aptasensor based on the immobilization of azido-aptamer onto electrografted binary film via click chemistry. Talanta.

[163] Villalonga A., Pérez-Calabuig A.M., Villalonga R. (2020). Electrochemical biosensors based on nucleic acid aptamers. Anal. Bioanal. Chem..

[164] Alawad A., Istamboulié G., Calas-Blanchard C., Noguer T. (2019). A reagentless aptasensor based on intrinsic aptamer redox activity for the detection of tetracycline in water. Sens. Actuators B Chem..

[165] Wang Q., Vasilescu A., Wang Q., Coffinier Y., Li M., Boukherroub R., Szunerits S. (2017). Electrophoretic Approach for the Simultaneous Deposition and Functionalization of Reduced Graphene Oxide Nanosheets with Diazonium Compounds: Application for Lysozyme Sensing in Serum. ACS Appl. Mater. Interfaces.

[166] Balamurugan S., Obubuafo A., Soper S.A., Spivak D.A. (2008). Surface immobilization methods for aptamer diagnostic applications. Anal. Bioanal. Chem..

[167] Simon L., Bognár Z., Gyurcsányi R.E. (2020). Finding the Optimal Surface Density of Aptamer Monolayers by SPR Imaging Detection-based Aptamer Microarrays. Electroanalysis.

[168] White R.J., Phares N., Lubin A.A., Xiao Y., Plaxco K. (2008). Optimization of Electrochemical Aptamer-Based Sensors via Optimization of Probe Packing Density and Surface Chemistry. Langmuir.

[169] Liu Y., Tuleouva N., Ramanculov E., Revzin A. (2010). Aptamer-Based Electrochemical Biosensor for Interferon Gamma Detection. Anal. Chem..

[170] Leroux Y.R., Hapiot P. (2013). Nanostructured Monolayers on Carbon Substrates Prepared by Electrografting of Protected Aryldiazonium Salts. Chem. Mater..

[171] Mishyn V., Aspermair P., Leroux Y., Happy H., Knoll W., Boukherroub R., Szunerits S. (2019). “Click” Chemistry on Gold Electrodes Modified with Reduced Graphene Oxide by Electrophoretic Deposition. Surfaces.

[172] Liu Y., Canoura J., Alkhamis O., Xiao Y. (2021). Immobilization Strategies for Enhancing Sensitivity of Electrochemical Aptamer-Based Sensors. ACS Appl. Mater. Interfaces.

[173] Gu Q., Nanney W., Cao H.H., Wang H., Ye T. (2018). Single Molecule Profiling of Molecular Recognition at a Model Electrochemical Biosensor. J. Am. Chem. Soc..

[174] Peyrin E. (2009). Nucleic acid aptamer molecular recognition principles and application in liquid chromatography and capillary electrophoresis. J. Sep. Sci..

[175] Chen H., Meisburger S.P., Pabit S.A., Sutton J.L., Webb W.W., Pollack L. (2012). Ionic strength-dependent persistence lengths of single-stranded RNA and DNA. Proc. Natl. Acad. Sci. USA.

[176] Palchetti I., Mascini M. (2012). Electrochemical nanomaterial-based nucleic acid aptasensors. Anal. Bioanal. Chem..

[177] Meirinho S.G., Dias L., Peres A., Rodrigues L.R. (2016). Voltammetric aptasensors for protein disease biomarkers detection: A review. Biotechnol. Adv..

[178] Xiao Y., Lubin A.A., Heeger A.J., Plaxco K. (2005). Label-Free Electronic Detection of Thrombin in Blood Serum by Using an Aptamer-Based Sensor. Angew. Chem. Int. Ed..

[179] Zejli H., Goud K.Y., Marty J.L. (2019). An electrochemical aptasensor based on polythiophene-3-carboxylic acid assisted methylene blue for aflatoxin B1 detection. Sens. Bio-Sens. Res..

[180] Wang Y., Gong C., Zhu Y., Wang Q., Geng L. (2021). Signal-on electrochemical aptasensor for sensitive detection of sulfamethazine based on carbon quantum dots/tungsten disulfide nanocomposites. Electrochim. Acta.

[181] Spring S., Goggins S., Frost C. (2021). Ratiometric Electrochemistry: Improving the Robustness, Reproducibility and Reliability of Biosensors. Molecules.

[182] Zhu C., Liu D., Li Y., Shen X., Ma S., Liu Y., You T. (2020). Ratiometric electrochemical aptasensor for ultrasensitive detection of Ochratoxin A based on a dual signal amplification strategy: Engineering the binding of methylene blue to DNA. Biosens. Bioelectron..

[183] Yang J., Hu Y., Li Y. (2019). Molecularly imprinted polymer-decorated signal on-off ratiometric electrochemical sensor for selective and robust dopamine detection. Biosens. Bioelectron..

[184] Zhu C., Liu D., Li Y., Ma S., Wang M., You T. (2021). Hairpin DNA assisted dual-ratiometric electrochemical aptasensor with high reliability and anti-interference ability for simultaneous detection of aflatoxin B1 and ochratoxin A. Biosens. Bioelectron..

[185] Jia F., Li Y., Gong Q., Liu D., Meng S., Zhu C., You T. (2022). A Simple Ratiometric Electrochemical Aptasensor Based on the Thionine–Graphene Nanocomposite for Ultrasensitive Detection of Aflatoxin B2 in Peanut and Peanut Oil. Chemosensors.

[186] Martin J.A., Chávez J.L., Chushak Y., Chapleau R., Hagen J., Kelley-Loughnane N. (2014). Tunable stringency aptamer selection and gold nanoparticle assay for detection of cortisol. Anal. Bioanal. Chem..

[187] Karuppaiah G., Velayutham J., Hansda S., Narayana N., Bhansali S., Manickam P. (2022). Towards the development of reagent-free and reusable electrochemical aptamer-based cortisol sensor. Bioelectrochemistry.

[188] Mugo S.M., Alberkant J., Bernstein N., Zenkina O.V. (2021). Flexible electrochemical aptasensor for cortisol detection in human sweat. Anal. Methods.

[189] Singh N.K., Chung S., Sveiven M., Hall D.A. (2021). Cortisol Detection in Undiluted Human Serum Using a Sensitive Electrochemical Structure-Switching Aptamer over an Antifouling Nanocomposite Layer. ACS Omega.

[190] Sun L., Zhao Q. (2018). Direct fluorescence anisotropy approach for aflatoxin B1 detection and affinity binding study by using single tetramethylrhodamine labeled aptamer. Talanta.

[191] Mehennaoui S., Poorahong S., Jimenez G.C., Siaj M. (2019). Selection of high affinity aptamer-ligand for dexamethasone and its electrochemical biosensor. Sci. Rep..

[192] Lim H.J., Kim A.-R., Yoon M.-Y., You Y., Chua B., Son A. (2018). Development of quantum dot aptasensor and its portable analyzer for the detection of di-2-ethylhexyl phthalate. Biosens. Bioelectron..

[193] Lee K., Gurudatt N., Heo W., Hyun K.-A., Jung H.-I. (2022). Ultrasensitive detection and risk assessment of di(2-ethylhexyl) phthalate migrated from daily-use plastic products using a nanostructured electrochemical aptasensor. Sens. Actuators B Chem..

[194] Stanciu L.A., Wei Q., Barui A.K., Mohammad N. (2021). Annual Review of Biomedical Engineering Recent Advances in Aptamer-Based Biosensors for Global Health Applications. Annu. Rev. Biomed. Eng..

